# Biocatalytic Parallel Interconnected Dynamic Asymmetric Disproportionation of α‐Substituted Aldehydes: Atom‐Efficient Access to Enantiopure (*S*)‐Profens and Profenols

**DOI:** 10.1002/adsc.201800541

**Published:** 2018-06-12

**Authors:** Erika Tassano, Kurt Faber, Mélanie Hall

**Affiliations:** ^1^ Department of Chemistry University of Graz Heinrichstrasse 28 8010 Graz Austria

**Keywords:** Alcohol dehydrogenase, asymmetric disproportionation, biocatalysis, Cannizzaro, design of experiments, profens

## Abstract

The biocatalytic asymmetric disproportionation of aldehydes catalyzed by horse liver alcohol dehydrogenase (HLADH) was assessed in detail on a series of racemic 2‐arylpropanals. Statistical optimization by means of design of experiments (DoE) allowed the identification of critical interdependencies between several reaction parameters and revealed a specific experimental window for reaching an ′optimal compromise′ in the reaction outcome. The biocatalytic system could be applied to a variety of 2‐arylpropanals and granted access in a redox‐neutral manner to enantioenriched (*S*)‐profens and profenols following a parallel interconnected dynamic asymmetric transformation (PIDAT). The reaction can be performed in aqueous buffer at ambient conditions, does not rely on a sacrificial co‐substrate, and requires only catalytic amounts of cofactor and a single enzyme. The high atom‐efficiency was exemplified by the conversion of 75 mM of *rac*‐2‐phenylpropanal with 0.03 mol% of HLADH in the presence of ∼0.013 eq. of oxidized nicotinamide adenine dinucleotide (NAD^+^), yielding 28.1 mM of (*S*)‐2‐phenylpropanol in 96% *ee* and 26.5 mM of (*S*)‐2‐phenylpropionic acid in 89% *ee*, in 73% overall conversion. Isolated yield of 62% was obtained on 100 mg‐scale, with intact enantiopurities.

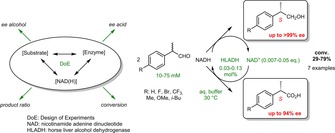

## Introduction

**Scheme 1:**
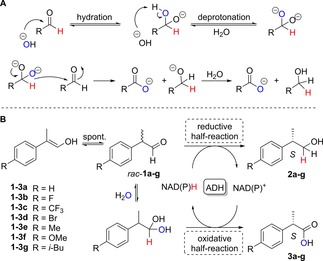
Disproportionation of A) non‐enolizable aldehydes in the base‐catalyzed Cannizzaro reaction^1^,^2^ and B) racemic 2‐arylpropanals (*rac*‐**1 a**–**g**) in the biocatalytic formal Cannizzaro reaction catalyzed by alcohol dehydrogenase (ADH).

The Cannizzaro reaction is a redox‐neutral disproportionation reaction, in which two molecules of an aldehyde undergo concurrent oxidation and reduction, yielding an equimolar mixture of corresponding alcohol and carboxylic acid.^1^ In the classical procedure, rather harsh conditions are required, such as use of a strong base (e. g. alkali metal hydroxide) and high temperature, which overall limit the protocol to non‐enolizable aldehydes. The generally accepted mechanism starts with the hydration of the aldehyde followed by deprotonation by a strong base; the generated unstable dianionic species then transfers a hydride directly onto a second aldehyde molecule, leading to a carboxylate and an alkoxide that gets protonated by the solvent to yield the alcohol (Scheme 1A).^2^ Various modifications have been implemented in the past decades, as for example, solvent‐free conditions,^3^ microwave irradiation,^4^ organo‐base mediation^5^ or Lewis acid catalysis.3a,^6^

We established a biocatalytic formal Cannizzaro reaction^7^ that relies on the concurrent oxidative and reductive activity of nicotinamide‐dependent alcohol dehydrogenases (ADHs) to run in an overall redox neutral fashion. In contrast to the chemical variant, the hydride transfer operates through the cofactor as a hydride shuttle (Scheme 1). Importantly, the oxidative half‐reaction proceeds on the hydrate form of the aldehyde,^8^ which – if the hydration is rate limiting – induces a lag in the coupling of the two redox reactions and thus furnishes a sub‐optimal ratio of products.^7^ The biocatalytic system exists therefore because of the oxidative catalytic activity of ADHs on geminal diols, which, although well‐studied,^9^ remains largely in the shadow of reductive processes.

Owing to the limited scope of the chemical reaction, cases of stereoselective Cannizzaro reaction are rare. Only a few intramolecular versions have been reported in literature to date, almost all applying arylglyoxals as starting material and exploiting Lewis acids complexed by sterically hindered chiral ligands.^10^ On the other hand, several ADHs showed significant enantioselectivity in the oxidation of α‐substituted aldehydes^11^ and we could exploit this feature to develop an intermolecular asymmetric biocatalytic formal Cannizzaro reaction employing a single ADH^7^ (Scheme 1B). From a synthetic standpoint, the system possesses attractive features: i) Overall redox‐neutral, the reaction requires only catalytic amounts of cofactor; ii) no sacrificial auxiliary substrate (coupled‐substrate approach) nor a second enzyme (coupled‐enzyme approach)^12^ are necessary to regenerate the nicotinamide cofactor; iii) starting from a single racemic compound, which spontaneously racemizes in the reaction buffer, two enantioenriched products (not necessarily homochiral) can be obtained *via* dynamic kinetic resolution. While this in theory should translate into highly atom‐efficient biotransformations, our initial set‐up applied to 2‐phenylpropanal (10 mM) still required ∼1 mol% catalyst (5 mg/mL) and 0.2 eq. of cofactor in form of a 1:1 NADH/NAD^+^ mixture to reach close to full conversion (Table 1, entry 6).^7^

**Table 1:** ADH‐mediated DKR approaches to enantiopure profen derivatives with cofactor recycling.

Entry^[a]^	[**1 a**] (mM)	[Product] (mM)	[NAD^+^] (mM/eq.)	NAD Recycling^[b]^	[ADH] (mg/mL)	TON_NAD_	TTN_ADH_	*ee*_product_ (*S*) %
118a	0.5	0.27 (**2 a**)	0.01/0.02	Coupled‐substrate EtOH^[c]^	0.01	44	1038	90 (**2 a**)
221b	5	3.7 (**2 a**)	0.05/0.01	Coupled‐substrate EtOH	n.r.^[d]^	74	n.d.	98 (**2 a**)
3^22^	5	4.9 (**2 a**)	0.1/0.02	Coupled‐substrate 1,4‐butanediol	0.1	49	1884	95 (**2 a**)
4^11^	5	2.4 (**3 a**)	2/0.4	Coupled‐enzyme NOX	20	1.2	5	50 (**3 a**)
5^11^	5	3.2 (**3 a**)	1/0.2	Coupled‐enzyme NOX	37.5	3.2	3	88 (**3 a**)
6^7^	10	5.2 (**2 a**) +4.5 (**3 a**)	1^[e]^/0.1	PIDAT	5	4.9^[f]^	76	93 (**2 a**) 88 (**3 a**)
7^[g]^	75	28.1 (**2 a**) +26.5 (**3 a**)	1^[h]^/0.01	PIDAT	1^[h]^	26.5^[i]^	2100	96 (**2 a**) 89 (**3 a**)

^[a]^ Data from literature, see references. ^[b]^ NOX: NAD(P)H oxidase; PIDAT: Parallel Interconnected Dynamic Asymmetric Transformation (one enzyme, no co‐substrate). ^[c]^ 10% THF as co‐solvent. ^[d]^ n.r. not reported (0.5 mU/mL on benzyl alcohol; enzyme obtained with 1.4 U per liter culture). ^[e]^ NADH/NAD^+^, 1:1, 1 mM each. ^[f]^ TON of each redox state of nicotinamide molecule (NADH/NAD^+^ pair). ^[g]^ 4 vol% MTBE. ^[h]^ Total amount (added in two equal portions over 24 h, total reaction time 48 h). ^[i]^ Each turn‐over converts two molecules of substrate. n.d. not determined.

Optimization of the reaction is associated with the necessary consideration of the four outputs: i) conversion level (highest possible), ii) ratio of products (close to 1:1), iii) enantiopurity of the alcohol and iv) enantiopurity of the carboxylic acid (both ideally >95%). Because several parameters affect the process simultaneously (e. g. amount of catalyst and cofactor, substrate concentration), the outcome of the biocatalytic disproportionation may be efficiently improved through statistical analysis. The use of chemometrics in biotechnology has been increasing over the last decade,^13^ and the employment of statistical experimental design^14^ in particular can contribute to process optimization while minimizing the number of experiments.^15^ For instance, the production of an oligosaccharide by yeast whole cells could be improved (in terms of yield and product concentration) by using a fractional factorial statistical design,^16^ while Kara *et al*. similarly applied this method to enhance the productivity of a bi‐enzymatic cascade for caprolactone synthesis.^17^ Herein, we present this approach to extend the asymmetric ADH‐catalyzed disproportionation to a range of racemic 2‐arylpropanals under synthetically relevant conditions, aiming at elevated substrate concentration and high atom‐efficiency (i. e. low catalyst‐ and cofactor loading along with high conversion). First, a range of experiments on the model substrate 2‐phenylpropanal was performed to identify the key elements impacting the four reaction outputs. Thereafter, a design of experiments (DoE) statistical analysis was conducted to determine potential (cross‐) interactions between the different reaction parameters that impact the overall outcome of the bioconversion. Finally, the scope of the reaction was evaluated with a particular focus on compounds yielding enantioenriched 2‐arylpropanols (profenols) – useful intermediates in medicinal chemistry and fragrances^18^ – and 2‐arylpropanoic acids (profens) – compounds belonging to an important subclass of NSAIDs (non‐steroidal anti‐inflammatory drugs) with pharmacological activity attributed to the (*S*)‐form.^19^

## Results and Discussion

### Analysis of the Reaction on Model Substrate 2‐Phenylpropanal (1 a)

**Figure 1:**
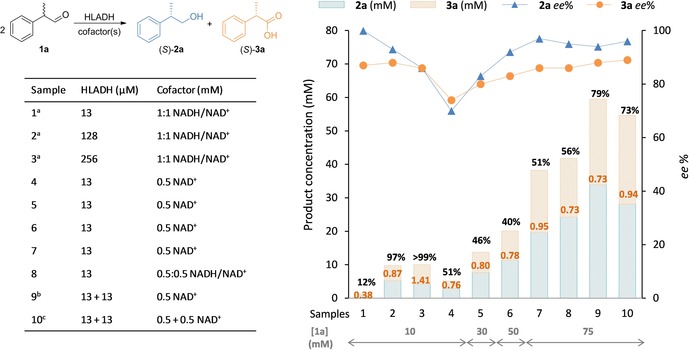
HLADH‐catalyzed asymmetric disproportionation of *rac*‐**1 a**. Conditions: phosphate buffer (50 mM, pH 7.5), 30 °C, 120 rpm, 24 h (see experimental section); entries 9–10: 4 vol% MTBE; 13 μM HLADH corresponds to 0.5 mg/mL; conversions (%) to (alcohol+acid) (bold black numbers on top of the bars) and *ee* values were determined by chiral GC analysis after product extraction (see Supporting Information); carboxylic acid to alcohol product ratio (bold orange numbers in the bars). ^a)^ Data from ref. 7. ^b)^ After 24 h, a second aliquot of enzyme was added; total reaction time 48 h. ^c)^ After 24 h, a second aliquot of enzyme and cofactor was added; total reaction time 48 h.

The disproportionation of **1 a** was previously evaluated with a panel of alcohol dehydrogenases (ADHs), and it was noted that horse liver ADH (HLADH) provided both (*S*)‐configurated products with high stereoselectivity.^7^ Under the tested conditions (0.5 mg/mL HLADH – ∼13 μM – and 0.1 eq. each of NADH and NAD^+^), HLADH showed a predominant reductive half‐reaction (ratio **3 a**/**2 a** 0.38) and a poor overall conversion (12%, Figure 1, sample 1). A 10‐fold increase in the biocatalyst amount (5 mg/mL, i. e. ∼1.3 mol%) led to higher conversion with modest erosion of the enantiopurity of the alcohol (Figure 1, sample 2). However, the high cofactor loading applied appeared in contradiction to the catalytic amount theoretically needed (i. e. only 1 equivalent of cofactor per enzyme due to internal recycling). Furthermore, we assumed that the oxidation state of the nicotinamide cofactor would play a crucial role to achieve a balanced ratio of products. Reducing the amount of NAD^+^ to 0.05 eq. at low enzyme concentration (13 μM) led to an unexpected 5‐fold increase in conversion level (Figure 1, sample 4) and a more balanced product ratio (**3 a**/**2 a** ∼0.76). Hence, providing only NAD^+^ seems key to achieving the latter and is likely associated with sufficient aldehyde hydration required for the first oxidative half‐reaction to take place (*vide infra*). However, a dramatic drop in the stereoselectivity of both half‐reactions was observed.

Next, the substrate loading was increased (Figure 1, samples 5–7). Remarkably, HLADH sustained aldehyde concentrations up to 75 mM, typically not well tolerated by enzymes due to the formation of Schiff bases with free amino groups of surface amino acids,^20^ which leads to protein deactivation. This translated into markedly improved product concentrations (19.6 mM **2 a** and 18.6 mM **3 a**), excellent enantioselectivity towards both alcohol and carboxylic acid [(*S*)‐**2 a** in 97% *ee* and (*S*)‐**3 a** in 86% *ee*] and well balanced product ratio (**3 a**/**2 a** 0.95).

Only modest increase in conversion along with out‐ of‐balance product ratio were observed with the pair of cofactors (Figure 1, sample 8). Collectively, the data suggest that combining higher substrate concentration (75 mM) with catalytic amounts of NAD^+^ (0.007 eq.) and low enzyme concentration (∼0.02 mol%) offers an excellent compromise to maximize all reaction outputs, i. e. high conversion, ideal product ratio and excellent stereocontrol in both redox reactions.

Finally, a further improvement of the overall conversion was achieved through the addition of fresh enzyme after 24 h (Figure 1, samples 9–10), with excellent stereocontrol retained in both half‐reactions. Moreover, a concomitant addition of NAD^+^ after 24 h (Figure 1, sample 10) proved to be crucial for a well‐balanced product ratio (73% overall conversion, with 28.1 mM of (*S*)‐**2 a** in 96% *ee* and 26.5 mM of (*S*)‐**3 a** in 89% *ee*). Both supplementations likely compensate for slow enzyme deactivation and (spontaneous) cofactor degradation over time, respectively.

The overall performance of the process favorably compares to related studies on the combination of ADH‐mediated dynamic kinetic resolution (DKR) of *rac*‐2‐arylpropanals, such as *rac*‐**1 a**, with cofactor recycling (Table 1). Reductive DKR21a was first developed in a coupled‐substrate approach using ethanol as co‐substrate.18a,21b While HLADH was limited to low substrate concentration (0.5 mM, Table 1, entry 1), ADH‐10 from *Sulfolobus solfataricus* was applied on a 5 mM scale and yielded (*S*)‐**2 a** in high enantiopurity with only 0.01 eq. NADH (Table 1, entry 2). In conjunction with 1,4‐butanediol as ′smart′ co‐substrate,^22^ the efficiency of HLADH could be increased on a 5 mM scale using 0.02 eq. NADH (98% conversion, Table 1, entry 3). Hollmann *et al*. then developed an oxidative variant based on a coupled‐enzyme approach, which relied on NAD(P)H oxidase (NOX) for cofactor recycling.^11^ Only a few cofactor turnovers could be reached at high NAD^+^ concentration and 5 mM **1 a**, which was attributed to non‐overlapping process windows of both enzymes (Table 1, entries 4–5). In this regard, the parallel interconnected dynamic asymmetric transformation (PIDAT)^23^ developed here is highly competitive among all known biocatalytic DKRs of **1 a**, with lowest cofactor concentration needed, highest product concentration and highest TTN of the biocatalyst reached.

The disproportionation of **1 a** was performed on a semi‐preparative scale (100 mg) according to the improved procedure (Figure 1, entry 10). After isolation and purification of the products, 31 mg of (*S*)‐**2 a** in 97% *ee* and 35 mg of (*S*)‐**3 a** in 86% *ee* (no racemization, data not shown) were obtained in an overall isolated yield of 62% and perfect product ratio of 1:1.03 (see experimental section for details).

### Design of Experiments

**Scheme 2:**
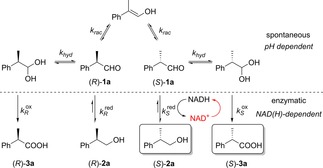
Equilibria and reactions involved in the asymmetric biocatalytic disproportionation of *rac*‐**1 a** to (*S*)‐**2 a** and (*S*)‐**3 a** (key controlling enzymatic event in red).

Besides the two redox half‐reactions, the system includes two equilibria involving the aldehyde substrate, i. e. racemization *via* keto‐enol tautomerism and hydration (Scheme 2). To reach full conversion of *rac*‐**1 a** to two enantiopure products, e. g. (*S*)‐**2 a** and (*S*)‐**3 a**, two main requirements must be fulfilled:

(1) $$k{}_{R}{}^{\mathrm{red}}\ll k{}_{S}{}^{\mathrm{red}}\leq k{}_{rac}\left(k{}_{R}{}^{\mathrm{red}}<k{}_{rac}<k{}_{S}{}^{\mathrm{red}}\;\mathrm{has}\;\mathrm{been}\;\mathrm{shown}\;\mathrm{possible}{}^{\left[24\right]}\right)$$

According to equation (**1**), a dynamic kinetic resolution (DKR) would establish a progressive and desired accumulation of (*S*)‐**2 a**.

(2) $$k{}_{R}{}^{\mathrm{ox}}\ll k{}_{S}{}^{\mathrm{ox}}<k{}_{hyd}<k{}_{rac}\;\left(\mathrm{ideal}:\;k{}_{S}{}^{\mathrm{ox}}+k{}_{hyd}\approx k{}_{S}{}^{\mathrm{red}}\right)$$

Since the oxidation is non‐reversible, the consumption of the hydrate form of (*S*)‐**1 a** controls the equilibria by pulling the overall system towards hydrate formation of the faster reacting enantiomer, which is the underlying reason for choosing the oxidized cofactor to initiate the reaction (red event, Scheme 2).^23^ Maximal efficiency is in theory attained when racemization is the most rapid event [equation (**2**)]. Increasing the enzyme concentration usually correlated with decreased stereoselectivity and it was speculated that the too slow racemization, combined with imperfect enzyme stereoselectivity, could not provide a steady pool of the faster reacting enantiomer (e. g. *k_rac_*<*k*_R_^ox^<*k*_S_^ox^).^11^,^21^

In order to sustain a constant ratio of products, the ideal system should possess a reductive half‐reaction rate for the desired enantiomer comparable to the overall rate of the hydration step and oxidative half‐reaction combined (*k*_S_^red^=*k*_S_^ox^+*k_hyd_*<*k_rac_*). Importantly, the low concentration of NAD^+^ (0.007–0.01 eq.) allows a tight control of the PIDAT as all steps are connected to the oxidative half‐reaction taking place first (Figure 1).

Given the intricacy of all steps involved (Scheme 2), a concomitant optimization of four responses (conversion level, ratio of products, enantiopurity of alcohol and carboxylic acid) appears challenging. A multivariate statistical analysis was thus performed by means of design of experiments (DoE), using Design‐Expert® from Stat‐Ease, Inc. As described above, three parameters (variables) were considered for the statistical analysis (Table 2): concentration of the enzyme (A), of the oxidized nicotinamide cofactor (B), and of the substrate (C), while temperature (30 °C), buffer (KPi, pH 7.5) and reaction time (24 h) were kept constant. The selected variables were employed for the development of a Central Composite Design (CCD), which is a Response Surface Method (RSM) that analyzes the variables at five different levels and which strength consists in the efficient evaluation of interactions and quadratic effects.^25^ CCD consists of a factorial design (2^*k*^, where *k* is the number of the parameters) augmented with a set of ′star points′ (2*k*, placed at the distance of ±α from the center point) to estimate the curvature. Considering the number of variables (*k*=3), duplicates and center points (n=4), the overall final number of experiments is 36 (2^*k*^+2*k*+*n*). The standard reaction conditions are described in the experimental section and for the entire experimental matrix and corresponding results, see the Supporting Information.

**Table 2:** Selected variables and relative levels in the Central Composite Design.

			Levels (coded)		
Factor^[a]^	Low (−1)	Central (0)	High (+1)	Axial (−α)^[b]^	Axial (+α)^[b]^
A [HLADH]	10	16	22	7	26
B [NAD^+^]	0.40	0.63	0.85	0.25	1.00
C [**1 a**]	25	48	70	10	85

^[a]^ A in μM, B and C in mM. ^[b]^ ′Star points′ were set at α=1.68179 to maintain rotability

Four different responses were evaluated: conversion (%), product ratio (**3 a**/**2 a**), *ee* of (*S*)‐**2 a** (%) and *ee* of (*S*)‐**3 a** (%). The collected data were analyzed by ANOVA, which assesses the significance of the effect of each parameter and their interactions (for the calculations, see Supporting Information). Multiple regression analysis provided the final reduced model equations, one for each specific response (**3**)–(**6**) [coefficients are given as coded values and significance levels were assessed by stars (*p≤0.1; **p≤0.05; *** *p*≤0.(3)]:

(3) $$\left(\mathrm{conversion}\right)=43.80+4.08\;A{}^{*}{}^{*}{}^{*}+1.57B{}^{*}{}^{*}{}^{*}-4.53C{}^{*}{}^{*}{}^{*}+2.21\mathrm{AC}{}^{*}{}^{*}{}^{*}+1.01\mathrm{BC}{}^{*}-1.56\;A{}^{2}{}^{*}{}^{*}{}^{*}-2.08C{}^{2}{}^{*}{}^{*}{}^{*}$$

(4) $$\left(3\;a/2\;a\right){}^{3}=0.4950+0.0197B{}^{*}+0.0413C{}^{*}{}^{*}{}^{*}-0.0408C{}^{2}{}^{*}{}^{*}{}^{*}$$

(5) $$\left(ee\;\left(S\right)\text{-}2\;a\right){}^{2}=7424.87-964.96\;A{}^{*}{}^{*}{}^{*}-407.09B{}^{*}{}^{*}{}^{*}+1480.24C{}^{*}{}^{*}{}^{*}+340.29\mathrm{AC}{}^{*}{}^{*}{}^{*}+\;92.90\mathrm{BC}{}^{*}{}^{*}+138.56B{}^{2}{}^{*}{}^{*}{}^{*}-440.29C{}^{2}{}^{*}{}^{*}{}^{*}$$

(6) $$\left(ee\;\left(S\right)\text{-}3\;a\right){}^{3}=571200-16442.8\;A{}^{*}{}^{*}{}^{*}-11175.59B{}^{*}{}^{*}+57493.75C{}^{*}{}^{*}{}^{*}+13803.96\mathrm{AC}{}^{*}{}^{*}-24716.31C{}^{2}{}^{*}{}^{*}{}^{*}$$

To validate the models and assess their predictive power, a defined set of experimental conditions was chosen (A=13 μM; B=0.5 mM; C=50 mM) and the models were applied to anticipate the outcome of the corresponding reaction. The experimental data perfectly matched with the prediction (Table 3), thereby validating the four equations. Given the strong variations in product enantiopurity reported under varying conditions (Figure 1 and Table 1), the predictive power of the statistical models applied to a biological system is remarkable. The equations and their relative perturbation plots, in which the sensitivity of a response to each significant factor (cross‐interactions not taken into account) is described graphically (Figure S2), were analyzed, bearing in mind that a steep slope or curvature correlates with a strong influence of the variable on the outcome (response). Remarkably, at least one significant quadratic term was included in all models [italic terms in (**3**)–(**6**)]: for instance, increasing the substrate concentration (C) suggested a substantial positive effect on the overall stereoselectivity of the reaction, as well as on the product ratio, but appears detrimental to the conversion. Moreover, C acted as the only significant variable able to control the product ratio, whereas the cofactor amount (B) had a strong influence on both conversion and enantiopurity of (*S*)‐**2 a**, however with opposite trend. Finally, increasing the enzyme concentration (A) dramatically affected the stereoselectivity, while having a positive influence on the conversion.

**Table 3:** Validation of the model by prediction of the four responses and corresponding experimental results at pre‐defined conditions.^[a]^

Response	Predicted	Experimental^[b]^	95% CI^[c]^
Conversion (%)	39	40	38–41
Ratio **3 a**/**2 a**	0.79	0.77	0.77–0.81
*ee* (*S*)‐**2 a** (%)	91	92	91–92
*ee* (*S*)‐**3 a** (%)	84	83	83–85

^[a]^ A=13 μM; B=0.5 mM; C=50 mM. ^[b]^ Mean of duplicate experiments. ^[c]^ Interval of confidence. Data from the prediction were rounded for clarity.

**Figure 2:**
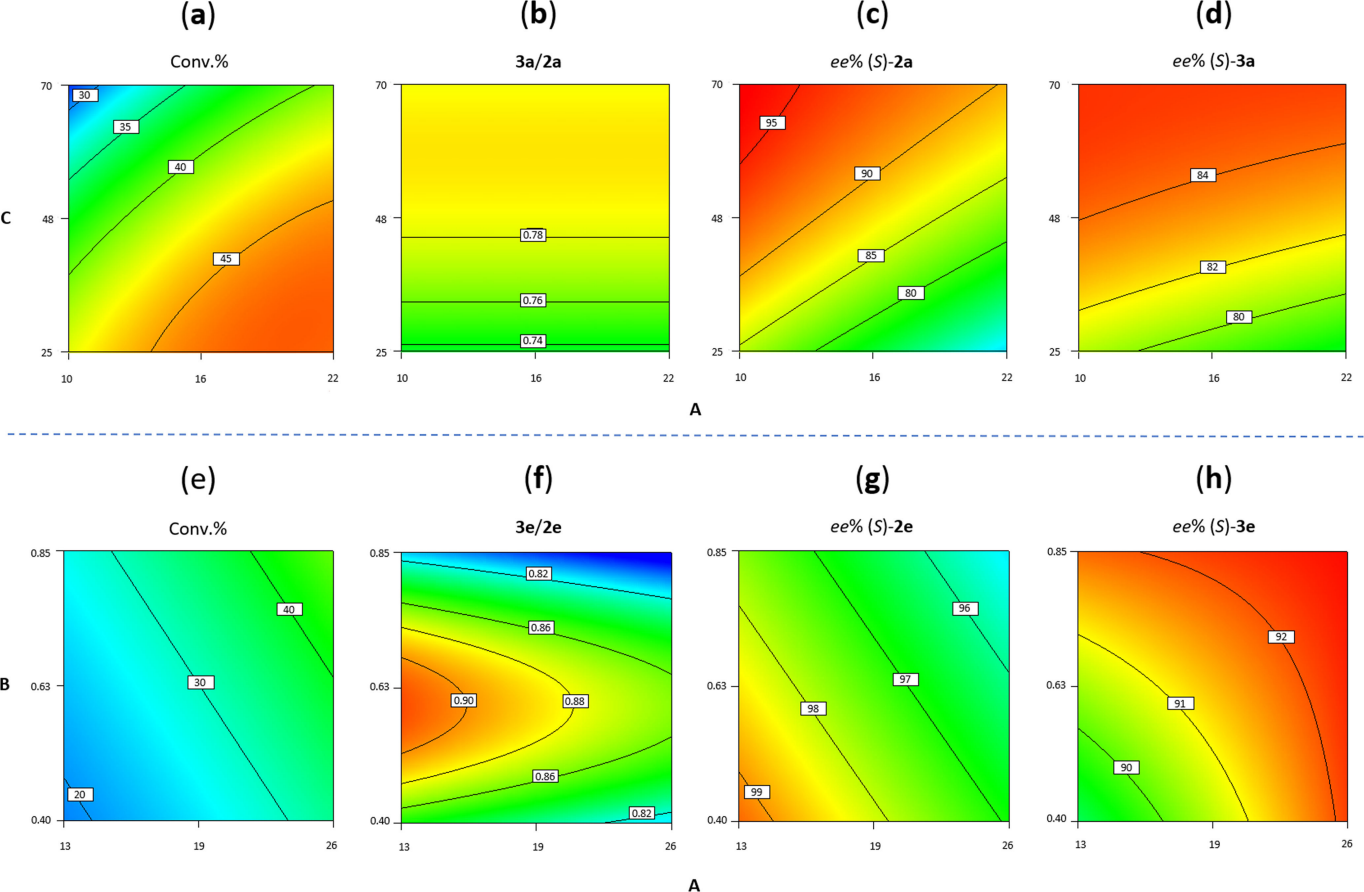
Contour plots (DesignExpert®) of all responses; warmer (colder) colors indicate higher (lower) values; A=[HLADH] (μM; x‐axis); B=[NAD^+^] or C=[**1 a**] (mM; y‐axis). Conversion of **1 a** (**a**), ratio **3 a**/**2 a** (**b**), *ee* of (*S*)‐**2 a** (**c**) and *ee* of (*S*)‐**3 a** (**d**); conversion of **1 e** (**e**), ratio **3 e**/**2 e** (**f**), *ee* of (*S*)‐**2 e** (**g**) and *ee* of (*S*)‐**3 e** (**h**). [NAD^+^] set to 0.5 mM in plots (**a**–**d**); [**1 e**] set to 30 mM in plots (**e**–**h**).

Successful optimization of the system requires above all identification of possible cross‐interactions between variables; these and identified trends can be visualized in the corresponding contour plots [NAD^+^ concentration set to 0.5 mM, Figure 2(**a**–**d**)]. Isolines parallel to the x‐axis in plot (**b**) illustrate the sole dependence of the ratio on C (substrate concentration). In the other three plots, the concurrent effect of both A and C (enzyme and aldehyde concentration, respectively) is depicted as diagonal isopleths: their curvature indicates existing cross‐interactions. Importantly, the impracticality of a simultaneous optimization of all four responses is thereby highlighted, and an optimum compromise must be sought instead. Contour plots obtained at fixed enzyme concentration (C=50 mM) revealed weaker interactions between A and B (Figure S3) as well as opposite behaviors on conversion and stereoselectivity.

**Figure 3:**
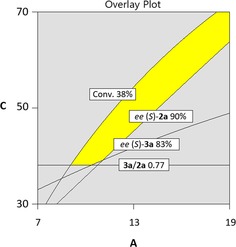
Overlay plot of the four responses (DesignExpert®); A=[HLADH] (μM; x‐axis); C=[**1 a**] (mM; y‐axis); the area where all required criteria are fulfilled at 0.5 mM NAD^+^ is highlighted in yellow (conversion >38%; ratio **3 a**/**2 a** >0.77, *ee* (*S*)‐**2 a** >90%; *ee* (*S*)‐**3 a** >83%).

In order to determine suitable ′sweet spots′ in which pre‐defined responses (desired reaction outcome) would be fulfilled, the optimization module of the software was used to screen for suitable combinations of parameters. Following constraints were applied: conversion >38%, ratio **3 a**/**2 a** >0.77, *ee* of (*S*)‐**2 a** >90% and *ee* of (*S*)‐**3 a** >83%. A section of the experimental domain matching these criteria at 0.5 mM NAD^+^ could be identified, thereby highlighting the rather narrow operational experimental window (yellow area, Figure 3); we could observe that the experimental conditions previously selected for the model validation were situated within the optimum range.

### Faced Centered Design Using 2‐(*p*‐tolyl)Propanal (1 e).

Encouraged by the results and the information obtained from the DoE analysis applied to the conversion of **1 a**, we adopted the same approach for the reaction with 2‐(*p*‐tolyl)propanal (**1 e**). The resulting model could again be experimentally validated at 95% confidence (Table S6 and Supporting Information). Interestingly, the resulting model equations for both conversion and *ee* of (*S*)‐**2 e** were composed only of linear terms and interactions, whereas those for ratio **3 e**/**2 e** and *ee* of (*S*)‐**3 e** required also ′pure quadratic′ terms. The effect of each single parameter was evaluated by analyzing the perturbation plots (Figure S4), which yielded the following observations: i) Increasing the enzyme concentration (A) had a positive effect on overall conversion – as generally expected – and enantiopurity of (*S*)‐**3 e**, but an adverse effect on product ratio and – unexpectedly – enantiopurity of (*S*)‐**2 e**, already hinting at a difficult optimization of the disproportionation of **1 e**; ii) the cofactor amount (B) had a similar influence, except on the ratio, where a marked curvature effect was observed; iii) the substrate concentration (C) was identified as a key parameter involved in interactions with an opposite effect to that of A and B.

While initial experiments indicated that it is possible to reach high conversion levels at low substrate concentration (Table S4, entries 7–8), the conserved excellent enantioselectivity in the oxidative half‐reaction was unfortunately accompanied by a decrease in the enantiopurity of (*S*)‐**2 e** and a product ratio in favor of the alcohol. The described general trends were far more evident from the contour plots, obtained this time at fixed substrate concentration ([**1 e**]=30 mM, Figure 2(**e**–**h**)). An exquisite stereoselectivity in the reductive half‐reaction was not only coupled with poor to moderate conversion, but the unlikelihood of a concurrent perfect enantioselectivity in both half‐reactions was also manifest, since the two optimum areas pointed in opposite directions [Figure 2(**g**–**h**)]. The quadratic effect of the cofactor amount on **3 e**/**2 e** was also evident, with slight deviations from the optimum value of B leading to out‐of‐balance product ratio [Figure 2(**f**)].

Taken together, the results of the DoE analysis highlight the practical difficulty of attaining optimization of all outputs of the disproportionation reaction and its strong dependence on the substrate type.

### Substrate Scope of the PIDAT

Finally, the substrate scope of the HLADH‐driven disproportionation reaction was investigated with emphasis on 2‐arylpropanals bearing different groups in the *p*‐position, as this substitution pattern is a common feature in the profen NSAID sub‐class. Varying substrate concentrations (10–50 mM) were employed to evaluate the enzyme tolerance and performance at higher aldehyde loading (Table 4). Based on the findings derived from the DoE analysis, NAD^+^ was added at 0.5 mM concentration.

**Table 4:** Substrate scope of HLADH‐catalyzed asymmetric disproportionation.^[a]^

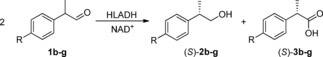						
Entry	R	[**1**] (mM)	Conv. (%)^[b]^	**3**/**2** ^[c]^	*ee* (*S*)‐**2** (%)	*ee* (*S*)‐**3** (%)
1	F	50	27	1.01	>99	94
2	(**1 b**)	30	32	0.89	99	94
3		10	34	0.82	>99	93
4	CF_3_	50	6	0.96	>99	77
5	(**1 c**)	30	12	0.93	>99	84
6		10	36	0.97	>99	90
7	Br	50	14	1.24	99	87
8	(**1 d**)	30	25	1.29	98	89
9		10	51	1.40	96	84
10	Me	50	11	0.86	99	87
11	(**1 e**)	30	18	0.88	99	89
12		10	29	0.91	99	90
13	MeO	50	19	1.01	99	93
14	(**1 f**)	30	31	0.99	99	93
15		10	71	0.98	98	93
16	*i*‐Bu	50	8	1.00	>99	84
17	(**1 g**)	30	16	1.09	99	90
18		10	31	1.09	91	90

^[a]^ Conditions: phosphate buffer (50 mM, pH 7.5), HLADH (0.5 mg/mL, ∼13 μM), 0.5 mM NAD^+^, 5 vol% MTBE as co‐solvent, 30 °C, 120 rpm, 24 h (see experimental section). ^[b]^ Conversions (to [alcohol+acid]) and *ee* values were determined by chiral GC analysis after product extraction (see Supporting Information). ^[c]^ Carboxylic acid to alcohol ratio.

Overall, the *p*‐substitution was well tolerated and HLADH catalyzed the asymmetric formal Cannizzaro reaction with exquisite stereocontrol in both half‐reactions on **1 b**–**g** [(*S*)‐**2** in 99–>99% *ee* and (*S*)‐**3** in 87–94% *ee*]. Compound **1 b** was particularly well accepted and yielded highest product amount (total 13.5 mM from 50 mM substrate, Table 4, entry 1) with perfect product ratio and highest enantiopurity for both products. Some general trends could be observed: product ratios were generally well‐balanced (except for **1 d**) and conversions increased with decreasing substrate concentration. Enantioselectivity toward (*S*)‐**2 b**–**g** was consistently excellent, indicating that the conditions applied were in this regard ideal. For most substrates, stereocontrol in the oxidation reaction tends to decrease upon increasing substrate concentration.

Interestingly, 2‐(4‐isobutylphenyl)propanal (**1 g**, precursor to ibuprofen) was also accepted (Table 4, entries 16–18): despite rather low conversion, ratio **3 g**/**2 g** was perfectly balanced, with ideal enantioselectivity. An atom‐efficient^26^ convergent enzymatic route to (*S*)‐ibuprofen based on HLADH‐mediated PIDAT of **1 g** is currently being developed and will be reported elsewhere. Altogether, no clear pattern in line with the electronic effects of the substitution could be identified, despite anticipation that electron‐withdrawing groups would be beneficial by enhancing racemization and, possibly, hydration^8^ rates. Indeed, TTN of HLADH was the highest with non‐substituted **1 a** (∼3 x 10^3^), followed by *p*‐fluoro‐derivative **1 b**, while bulky compound **1 c** led to the lowest value. *p*‐Bromo‐compound **1 d** and *p*‐methoxy‐compound **1 f** gave intermediate TTNs (Table S7).

## Conclusion

The biocatalytic asymmetric formal Cannizzaro reaction of α‐substituted aldehydes catalyzed by HLADH was investigated in detail, with particular attention to enantioselective formation of both alcohol and carboxylic acid products. In order to develop this intricate reaction to synthetically relevant conditions, aiming at high product concentration and perfect stereoselectivity, a statistical investigation by means of design of experiments (DoE) was performed. This provided a global understanding of the system and highlighted interactions between the parameters involved in the reaction. While a common optimum cannot be attained, an excellent compromise between conversion, product ratio and stereocontrol of both concurrent reactions was achieved at 75 mM concentration of aldehyde, yielding products in up to 99% *ee*. The transformation proceeds in an atom‐efficient manner, does not need a sacrificial co‐substrate and relies on a single enzyme and catalytic amounts of oxidized cofactor (max. 0.05 eq.). In 100 mg‐scale transformation, this translated into an effective requirement of 2 mol% NAD^+^ related to the amount of isolated products. A range of 2‐arylpropanals bearing substituents in the *p*‐position were converted to corresponding enantioenriched (*S*)‐′profenols′ and ′profens′, thereby highlighting the synthetic potential of the redox‐neutral^27^ disproportionation. While some substrates are still posing a challenge in terms of reactivity, the use of other ADHs could improve the reaction output. Promising remains the exquisite stereocontrol of the asymmetric dismutation reaction, which is achieved through concurrent oxidative and reductive reactions regulated by the oxidized cofactor acting as connector and applied in catalytic quantity. In several cases, HLADH appears well‐suited to install the stereochemistry with high precision in the final two products at almost ′no cost′.^28^ Further studies on practical applications of the parallel interconnected dynamic asymmetric transformation (PIDAT) for stereoselective synthesis are currently underway in our laboratories.

## **Experimental Section**

### General information

Chemical reagents and solvents were used as received from commercial sources. *Rac*‐**1 b**–**g** and reference compounds (*rac*‐ and (*S*)‐**2 b**–**g** and *rac*‐ and (*S*)‐**3 b**–**g**) were prepared according to modified procedures reported in literature, and fully characterized (see Supporting Information). Absolute configuration of enantiopure reference compounds was determined by comparison of experimental optical rotation values with literature data. Absolute stereochemistry of products from the biotransformations was assigned by chiral GC analysis by comparison with elution profiles of available racemic and corresponding enantiopure reference compounds (see Supporting Information).

### Cloning and Overexpression of HLADH

For expression of HLADH (E‐isoenzyme of ADH from *Equus caballus* forming dimer EE,^29^ cloned in pET28a with *N*‐terminal His‐tag), *E. coli* BL21 (DE3) cells transformed with pEG 54 plasmid were grown overnight at 30 °C and 120 rpm in a 50 mL Sarstedt tube containing LB/Kan medium (10 mL, 50 μg/mL kanamycin). LB/Kan medium (330 mL, 50 μg/mL kanamycin) in 1 L baffled shaking flasks was inoculated with 2 mL of the ONC. These cultures were shaken at 25 °C and 120 rpm for 4.5 h (OD600=0.6). IPTG solution (165 μL of a 1 M stock solution, 0.5 mM final concentration) was added. The cultures were shaken overnight at 25 °C and 120 rpm. Cells from 2 L of culture were harvested by centrifugation (4000 rpm, 20 min, 4 °C), washed with phosphate buffer A (50 mM, pH 7.5), centrifuged again (4000 rpm, 20 min, 4 °C) and resuspended in buffer A containing 20 mM imidazole. After sonication (Branson Sonifier 250; 3 min, 30% amplitude, 1 sec pulse, 4 sec pause), the disrupted cells were centrifuged (17000 rpm, 20 min, 4 °C). The supernatant was transferred through a fluted filter into a 50 mL Sarstedt tube, loaded on a HisTrap^TM^ FF column (5 mL), and washed with 20 column volumes of buffer A containing 20 mM imidazole. The column was washed with buffer A containing 150 mM imidazole (10 column volumes). The protein was eluted from the column with buffer A containing 300 mM imidazole (seven column volumes). Possible residual proteins were washed from the column with buffer A containing 500 mM imidazole (five column volumes). After SDS‐PAGE (Figure S1), fractions containing highly pure HLADH were combined, desalted overnight at 4 °C in a dialysis tube submerged into buffer A (4 L), freeze‐dried and stored at −21 °C.

### Standard Procedure for Disproportionation Reactions (PIDAT)

Enzymatic reactions were run as follows: an aliquot of purified HLADH was added to a phosphate buffer solution (final volume 1 mL, 50 mM, pH 7.5) containing *rac*‐**1 a**–**g** and the nicotinamide cofactor(s). The reaction mixture was incubated at 30 °C and 120 rpm. After 24 h, products were extracted with EtOAc (300 μL), the aqueous phase was acidified with HCl (100 μL, 3 N) and extracted again with EtOAc (300 μL). The combined organic fractions were dried over Na_2_SO_4_ and analyzed by chiral GC to determine both conversion (using calibration lines) and enantiomeric excesses of both products. For disproportionation reactions in presence of co‐solvent (**1 b**–**g**), MTBE was present in the buffer in 5 vol%; the workup was performed as above.

### Preparative Scale Disproportionation of 1 a

The reaction was performed on 100 mg **1 a** according to the procedure developed during the optimization phase (Figure 1, entry 10). Briefly, 100 mg of *rac*‐**1 a** (0.75 mmol) added from a stock solution in MTBE (4 vol% final concentration), 5 mg of freshly purified HLADH supplemented with ZnCl_2_ (100 μM final concentration) and 3 mg of NAD^+^ (5 μmol) were incubated at 30 °C and 120 rpm in 10 mL phosphate buffer (50 mM, pH 7.5). After 24 h, a second aliquot of both enzyme preparation (5 mg) and fresh cofactor (3 mg) were added, and the incubation was continued for 24 h. The mixture was then extracted with EtOAc and the solvent evaporated under vacuum. The crude was purified by flash chromatography (cyclohexane/EtOAc 8:2), obtaining 31 mg (0.23 mmol) of (*S*)‐**2 a**, in 97% *ee* (determined by chiral GC). ^1^H NMR (300 MHz, CDCl_3_): δ=7.38–7.19 (m, 5H), 3.70 (d, *J*=6.8 Hz, 2H), 2.95 (h, *J*=6.9 Hz, 1H), 1.46 (bs, 1H), 1.28 (d, *J*=7.0 Hz, 3H); NMR data in agreement with the literature.^30^ The aqueous phase was acidified with 3 N HCl and extracted with EtOAc; evaporating the solvent yielded 35 mg (0.23 mmol) of pure (*S*)‐**3 a** in 86% *ee* (determined by chiral GC). ^1^H NMR (300 MHz, CDCl_3_): δ=7.61–7.25 (m, 5H), 3.74 (q, *J*=7.2 Hz, 1H), 1.52 (d, *J*=7.2 Hz, 3H); NMR data in agreement with the literature.^31^ Overall isolated yield: 62%; ratio **3 a**/**2 a**: 1.03.

## Supporting information

As a service to our authors and readers, this journal provides supporting information supplied by the authors. Such materials are peer reviewed and may be re‐organized for online delivery, but are not copy‐edited or typeset. Technical support issues arising from supporting information (other than missing files) should be addressed to the authors.

Supplementary
