# Supplementary material for: Biocatalytic Parallel Interconnected Dynamic Asymmetric Disproportionation of α‐Substituted Aldehydes: Atom‐Efficient Access to Enantiopure (S)‐Profens and Profenols
Source: Adv Synth Catal. 2018 Jun 12;360(14):2742–51. doi: 10.1002/adsc.201800541 (PMC6099231; doi:10.1002/adsc.201800541)

Advanced Synthesis & Catalysis

Supporting Information

Supporting information

Biocatalytic Parallel Interconnected Dynamic Asymmetric Disproportionation of α-Substituted Aldehydes: Atom-Efficient Access to Enantiopure (*S*)-Profens and Profenols

Erika Tassano, Kurt Faber and Mélanie Hall*

Department of Chemistry, University of Graz, Heinrichstrasse 28, 8010 Graz, Austria

*corresponding author: melanie.hall@uni-graz.at

Table of contents

1. General methods S2
2. SDS page from purification of HLADH S3
3. DoE Analyses S3
   The design of experiments (DoE) was performed using Design-Expert® from Stat-Ease, Inc (version 11). .. S3
   Central Composite Design (CCD) with model substrate 2-phenylpropanal (**1a**) S3
   Face Centered Design (FCD) with 2-(4-methylphenyl)propanal (**1e**) S7
4. HLADH total turnover number S12
5. Synthesis of substrates and reference compounds S12
   General procedure for the synthesis of racemic aldehydes **1b-d** S12
   Synthesis of reference 2-(4-isobutylphenyl)propan-1-ol (**2g**) and 2-(4-isobutylphenyl)propanal (**1g**) S14
   General procedure for the synthesis of racemic alcohols **2b-f** S14
   General procedure for the synthesis of racemic acids **3b-f** S15
   Determination of the absolute configuration S16
   Typical enzymatic protocol for the synthesis of reference (*S*)-**2b-g** S16
   General oxidation protocol for the synthesis of reference (*S*)-**3b-f** S17
6. Chiral GC analyses S18

Analytical data for 2-phenylpropanal (**1a**) purchased S18
Analytical data for 2-phenylpropan-1-ol (**2a**)........ S18
Analytical data for 2-phenylpropanoic acid (**3a**)........ S19
Analytical data for 2-(4-fluorophenyl)propanal (**1b**)........ S19
Analytical data for 2-(4-fluorophenyl)propan-1-ol (**2b**)........ S19
Analytical data for 2-(4-fluorophenyl)propanoic acid (**3b**) S20
Analytical data for 2-(4-(trifluoromethyl)phenyl)propanal (**1c**)........ S21
Analytical data for 2-(4-(trifluoromethyl)phenyl)propan-1-ol (**2c**) S21
Analytical data for 2-(4-(trifluoromethyl)phenyl)propanoic acid (**3c**) S22
Analytical data for 2-(4-bromophenyl)propanal (**1d**)........ S22
Analytical data for 2-(4-bromophenyl)propan-1-ol (**2d**)........ S23
Analytical data for 2-(4-bromophenyl)propanoic acid (**3d**) S23
Analytical data for 2-(4-methylphenyl)propanal (**1e**) S24
Analytical data for 2-(4-methylphenyl)propan-1-ol (**2e**)........ S24
Analytical data for 2-(4-methylphenyl)propanoic acid (**3e**)........ S25
Analytical data for 2-(4-methoxyphenyl)propanal (**1f**) S25
Analytical data for 2-(4-methoxyphenyl)propan-1-ol (**2f**) S26
Analytical data for 2-(4-methoxyphenyl)propanoic acid (**3f**)........ S26
Analytical data 2-(4-isobutylphenyl)propanal (**1g**) S27
Analytical data 2-(4-isobutylphenyl)propan-1-ol (**2g**)........ S27
Analytical data for 2-(4-isobutylphenyl)propanoic acid (**3g**)........ S28

7. References........ S28

8. ^{1}H NMR spectra S30

1. General methods

Chemical reagents employed in the synthesis of starting and reference materials were purchased from Alfa-Aesar and Sigma-Aldrich. TLC analyses were performed on Merck Silica Gel 60 F_{254} precoated plates and visualized under UV light (λ = 254 nm), stained with Hanessian stain [dipping into a solution of $(NH_4)_4MoO_4·4H_2O$ (21 g) and $Ce(SO_4)_2·4H_2O$ (1 g) in H_2SO_4 (31 mL) and H_2O (469 mL) and warming] and with bromocresol green stain (solution of 0.1 g bromocresol green in 500 mL ethanol and 5 mL 0.1 N NaOH). Petroleum ether (40–60 °C) is abbreviated PE. Organic extracts were always dried with Na_2SO_4 and filtered before evaporation of the solvent under reduced pressure. Column chromatography was done with the 'flash' methodology[1] by using 220–400 mesh silica. NMR spectra were measured on a Bruker Avance III 300 MHz NMR spectrometer. Chemical shifts are reported in ppm relative to TMS (δ = 0.00 ppm) and coupling constants (J) in Hertz (Hz). Measurements of optical rotation were performed at 590 nm with a PerkinElmer 241 polarimeter. GC-MS analyses were performed on an Agilent 7890A GC system, equipped with an Agilent 5973 mass selective detector and an Agilent HP-5MS column (30 m x 0.25 mm x 0.25 µm).

2. SDS page from purification of HLADH

Figure S1: SDS page from purification of HLADH: 1 – supernatant after sonication; 2 – fraction from the loading phase; 3 – washing fraction; 4 – standard (PageRuler^{TM}, 10 to 180 kDa); 5 to 12 - elution fractions.

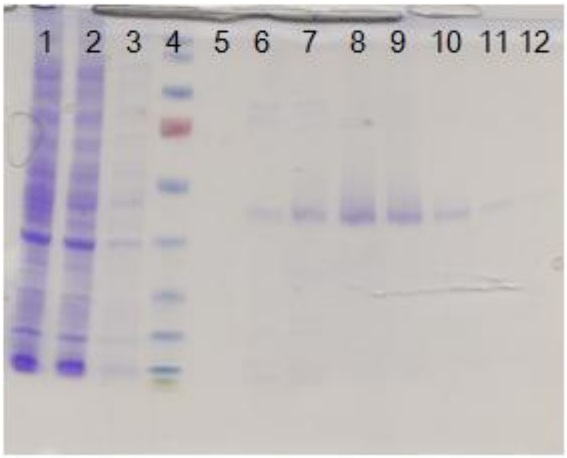

3. DoE Analyses

The design of experiments (DoE) was performed using Design-Expert® from Stat-Ease, Inc (version 11).

<u>Central Composite Design (CCD) with model substrate 2-phenylpropanal (1a)</u>

Table S1: experimental matrix of the CCD used in this study and results of the corresponding experiments (Design-Expert®)

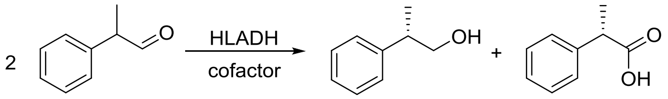

Entry	space type	A HLADH [µM]	B NAD^+ [mM]	C **1a** [mM]	Conv. %	Ratio **3a/2a**	*ee* (*S*)-**2a** %	*ee* (*S*)-**3a** %
1	factorial	10	0.40	25	45	0.71	86	84
2	factorial	10	0.40	25	45	0.79	86	82
3	factorial	22	0.40	25	48	0.67	70	80
4	factorial	22	0.40	25	48	0.70	69	79
5	factorial	10	0.85	25	46	0.75	81	81
6	factorial	10	0.85	25	47	0.78	81	81
7	factorial	22	0.85	25	48	0.74	63	77
8	factorial	22	0.85	25	48	0.74	63	78

9	factorial	10	0.40	70	27	0.77	96	86
10	factorial	10	0.40	70	27	0.77	96	84
11	factorial	22	0.40	70	36	0.70	91	83
12	factorial	22	0.40	70	39	0.75	91	85
13	factorial	10	0.85	70	29	0.75	95	85
14	factorial	10	0.85	70	32	0.81	95	82
15	factorial	22	0.85	70	43	0.75	87	84
16	factorial	22	0.85	70	43	0.79	87	84
17	axial	7	0.63	48	31	0.78	96	84
18	axial	7	0.63	48	30	0.81	96	84
19	axial	26	0.63	48	47	0.80	75	81
20	axial	26	0.63	48	47	0.81	77	82
21	axial	16	0.25	48	39	0.81	94	84
22	axial	16	0.25	48	40	0.80	92	84
23	axial	16	1.00	48	46	0.81	84	82
24	axial	16	1.00	48	45	0.83	84	82
25	axial	16	0.63	10	41	0.69	60	71
26	axial	16	0.63	10	40	0.66	60	70
27	axial	16	0.63	85	33	0.82	94	85
28	axial	16	0.63	85	34	0.81	94	85
29	center	16	0.63	48	45	0.78	86	83
30	center	16	0.63	48	46	0.80	85	82
31	center	16	0.63	48	43	0.78	86	82
32	center	16	0.63	48	44	0.81	86	82
33	center	16	0.63	48	44	0.83	87	83
34	center	16	0.63	48	44	0.82	86	82
35	center	16	0.63	48	43	0.81	86	82
36	center	16	0.63	48	45	0.76	87	82

Table S2: ANOVA results and coefficient validation for the four responses (Design-Expert®)

Conversion

Source	Sum of squares	Degrees of freedom	Mean square	F value	p value
Model	1329.17	7	189.88	35.76	< 0.0001
A	453.70	1	453.70	85.45	< 0.0001
B	67.18	1	67.18	12.65	0.0014
C	559.80	1	559.80	105.44	< 0.0001
AC	78.14	1	78.14	14.72	0.0007
BC	16.35	1	16.35	3.08	0.0902
A^2	64.45	1	64.45	12.14	0.0016
C^2	114.70	1	114.70	21.60	< 0.0001
Pure error	15.65	21	0.7451		
Cor total	1477.83	35			

Final equation (coded factors): (conv.%) = $43.80 + 4.08A + 1.57B – 4.53C + 2.21\,AC + 1.01BC – 1.56A^2 - 2.08C^2$

**R^2**	0.8994
**Adjusted R^2**	0.8743
**Predicted R^2**	0.8099
**Adeq Precision**	18.7275

Ratio **3a/2a**

Source	Sum of squares	Degrees of freedom	Mean square	F value	p value
Model	0.1027	3	0.0342	9.75	0.0001
B	0.0106	1	0.0106	3.03	0.0912
C	0.0465	1	0.0465	13.26	0.0009
C^2	0.0455	1	0.0455	12.97	0.0011
Pure error	0.0397	21	0.0019		
Cor total	0.2150	35			

Final equation (coded factors): $(\text{ratio})^3 = 0.4950 + 0.0197B + 0.0413C - 0.0408C^2$

**R^2**	0.4777
**Adjusted R^2**	0.4287
**Predicted R^2**	0.2918
**Adeq Precision**	11.0479

ee (*S*)-**2a**

Source	Sum of squares	Degrees of freedom	Mean square	F value	p value
Model	9.818E+07	7	1.403E+07	554.67	< 0.0001
A	2.543E+07	1	2.543E+07	1005.75	< 0.0001
B	4.526E+06	1	4.526E+06	179.00	< 0.0001
C	5.985E+07	1	5.985E+07	2366.66	< 0.0001
AC	1.853E+06	1	1.853E+06	73.27	< 0.0001
BC	1.381E+05	1	1.381E+05	5.46	0.0268
B^2	5.074E+05	1	5.074E+05	20.07	0.0001
C^2	5.129E+06	1	5.129E+06	202.83	< 0.0001
Pure error	2.185E+05	21	10403.42		
Cor total	9.889E+07	35			

Final equation (coded factors): (*ee* **2a**)^2 = 7424.87 – 964.96A – 407.09B + 1480.24C + 340.29AC + 92.90BC + 138.56B^2 – 440.29C^2

**R^2**	0.9928
**Adjusted R^2**	0.9911
**Predicted R^2**	0.9870
**Adeq Precision**	78.0660

ee (*S*)-**3a**

Source	Sum of squares	Degrees of freedom	Mean square	F value	p value
Model	1.208E+11	5	2.416E+10	39.10	< 0.0001
A	7.385E+09	1	7.385E+09	11.95	0.0017
B	3.411E+09	1	3.411E+09	5.52	0.0256
C	9.029E+10	1	9.029E+10	146.13	< 0.0001
AC	3.049E+09	1	3.049E+09	4.93	0.0340
C^2	1.666E+10	1	1.666E+10	26.97	< 0.0001
Pure error	4.531E+09	21	2.158E+08		
Cor total	1.393E+11	35			

Final equation (coded factors): (*ee* **3a**)^3 = 571200 – 16442.80A – 11175.59B + 57493.75C + 13803.96AC – 24716.31C^2

**R^2**	0.8670
**Adjusted R^2**	0.8448
**Predicted R^2**	0.7760
**Adeq Precision**	21.0089

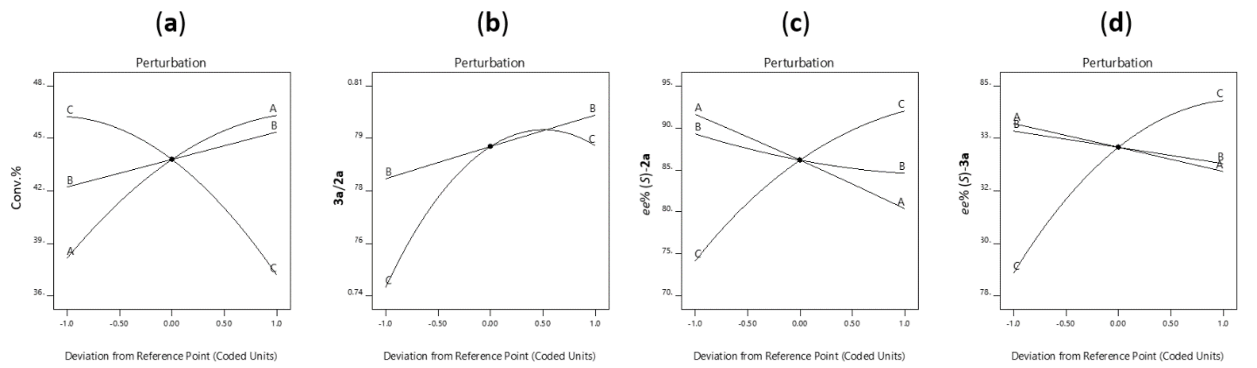

Figure S2: perturbation plots (Design-Expert®) of the four responses: conversion % (**a**), ratio **3a/2a** (**b**) *ee* of (*S*)-**2a** (**c**) and *ee* of (*S*)-**3a** (**d**).

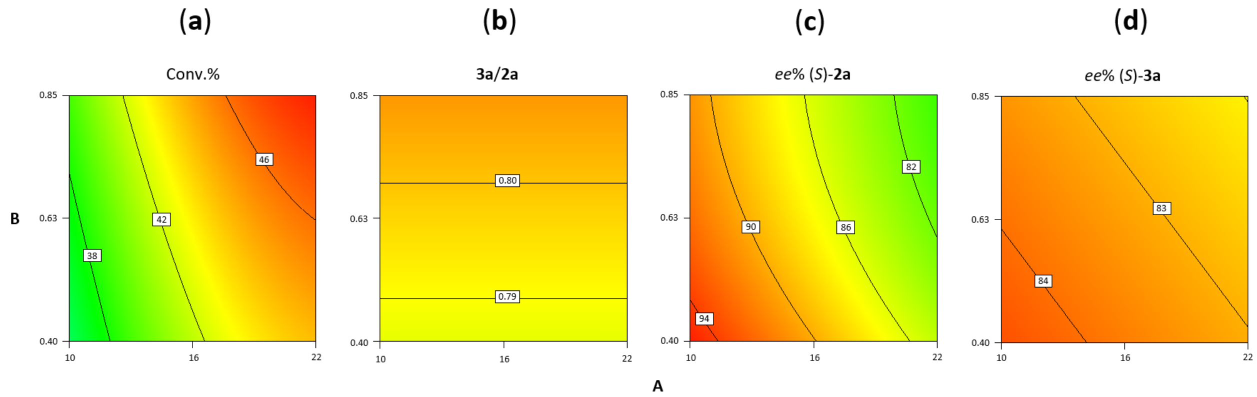

Figure S3: contour plots (Design-Expert®) for the responses: conversion % (**a**), ratio **3a/2a** (**b**), *ee* of (*S*)-**2a** (**c**) and *ee* of (*S*)-**3a** (**d**); warmer (colder) colors indicate higher (lower) values; parameters: A = [HLADH] (μM; x axis); B = [NAD^+] (mM; y axis); [**1a**] set to 50 mM.

Face Centered Design (FCD) with 2-(4-methylphenyl)propanal (1e)

The reported design was performed in two different runs, using two batches of substrate. Therefore, a blocking was implemented: experiments were split into two blocks, the first comprising the factorial points (with replicates), the latter composed by the axial and the center points. The selected parameters and their levels are reported in Table S3; temperature (30 °C) and reaction time (24 hours) were kept constant.

Table S3: selected variables and relative levels in the FCD

	Factor	Unit	Levels (coded)				
			Low (-1)	Central (0)	High (+1)	Axial (-α)[a]	Axial (+α)[a]
A	HLADH	μM	13	19.5	26	13	26
B	NAD^+	mM	0.50	0.75	1.00	0.50	1.00
C	Substrate	mM	10	30	50	10	50

[a] 'Star points' set at α = 1.

Table S4: experimental matrix of the FCD and corresponding results of the experiments (Design-Expert®)

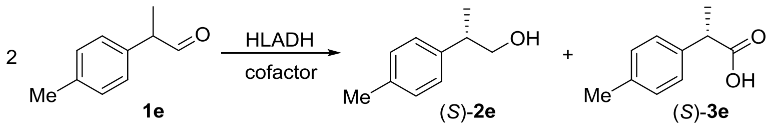

Entry	block	space type	A HLADH [µM]	B NAD^+ [mM]	C **1e** [mM]	Conv. (%)	Ratio **3e/2e**	*ee* (*S*)-**2e** %	*ee* (*S*)-**3e** %
1	A	factorial	13	0.5	10	32	0.91	99	90
2	A	factorial	13	0.5	10	26	0.90	99	90
3	A	factorial	26	0.5	10	63	0.82	95	91
4	A	factorial	26	0.5	10	34	0.81	97	92
5	A	factorial	13	1.0	10	48	0.83	94	92
6	A	factorial	13	1.0	10	49	0.80	98	92
7	A	factorial	26	1.0	10	69	0.78	94	92
8	A	factorial	26	1.0	10	72	0.80	94	92
9	A	factorial	13	0.5	50	11	0.86	100	87
10	A	factorial	13	0.5	50	11	0.86	100	87
11	A	factorial	26	0.5	50	25	0.78	97	91
12	A	factorial	26	0.5	50	26	0.87	97	92
13	A	factorial	13	1.0	50	18	0.87	98	92
14	A	factorial	13	1.0	50	18	0.81	98	90
15	A	factorial	26	1.0	50	30	0.85	97	92
16	A	factorial	26	1.0	50	30	0.80	96	93
17	B	axial	13	0.75	30	20	0.79	99	90
18	B	axial	26	0.75	30	37	0.87	97	91
19	B	axial	19.5	0.50	30	29	0.80	97	91
20	B	axial	19.5	1.0	30	32	0.78	98	91
21	B	axial	19.5	0.75	10	50	0.92	97	90
22	B	axial	19.5	0.75	50	20	0.87	98	89
23	B	center	19.5	0.75	30	31	0.85	97	90
24	B	center	19.5	0.75	30	23	0.83	96	90

Table S5: ANOVA results and coefficient validation for the four responses (Design-Expert®). In the reported equations coefficients are given as coded values and significance levels were assessed by stars (* $p \leq 0.1$; ** $p \leq 0.05$; *** $p \leq 0.01$)

Conversion

Source	Sum of squares	Degrees of freedom	Mean square	F value	p value
Block	0.0000	1	0.0000		
Model	0.0517	5	0.0103	69.78	< 0.0001
A	0.0136	1	0.0136	91.68	< 0.0001
B	0.0055	1	0.0055	37.03	< 0.0001
C	0.0294	1	0.0294	198.59	< 0.0001
AB	0.0010	1	0.0010	6.91	0.0176
AC	0.0022	1	0.0022	14.67	0.0013
Pure error	0.0016	9	0.0002		
Cor total	0.0542	23			

Final equation (coded factors): 1/Sqrt(conv.%) = + 0.1887 – 0.0275A*** – 0.0175B*** + 0.0404C*** + 0.0080AB** – 0.0117AC***

**R^2**	0.9535
**Adjusted R^2**	0.9399
**Predicted R^2**	0.9065
**Adeq Precision**	25.9704

Ratio **3e**/**2e**

Source	Sum of squares	Degrees of freedom	Mean square	F value	p value
Block	0.0808	1	0.0808		
Model	0.9412	3	0.3137	12.52	< 0.0001
A	0.3007	1	0.3007	12.00	0.0026
B	0.3014	1	0.3014	12.03	0.0026
B^2	0.3392	1	0.3392	13.54	0.0016
Pure error	0.2283	9	0.0254		
Cor total	1.50	23			

Final equation (coded factors): $(\text{ratio})^{-3}$ = 1.42 + 0.1293A*** + 0.1294B*** + 0.4755B^2***

**R^2**	0.6641
**Adjusted R^2**	0.6111
**Predicted R^2**	0.4929
**Adeq Precision**	10.1623

ee (*S*)-**2e**

Source	Sum of squares	Degrees of freedom	Mean square	F value	p value
Block	4.210E+08	1	4.210E+08		
Model	4.170E+10	4	1.043E+10	15.49	< 0.0001
A	2.120E+10	1	2.120E+10	31.49	< 0.0001
B	9.914E+09	1	9.914E+09	14.73	0.0012
C	8.472E+09	1	8.472E+09	12.59	0.0023
BC	2.122E+09	1	2.122E+09	3.15	0.0927
Pure error	6.870E+09	9	7.633E+08		
Cor total	5.424E+10	23			

Final equation (coded factors): $(ee\ \mathbf{2e})^3$ = 921400 – 34316.57A*** – 23468.85B*** + 21694.61C*** + 11514.96BC*

**R^2**	0.7749
**Adjusted R^2**	0.7249
**Predicted R^2**	0.5966
**Adeq Precision**	12.2541

ee (*S*)-**3e**

Source	Sum of squares	Degrees of freedom	Mean square	F value	p value
Block	1.271E+09	1	1.271E+09		
Model	2.723E+10	6	4.539E+09	15.78	< 0.0001
A	9.644E+09	1	9.644E+09	33.53	< 0.0001
B	6.973E+09	1	6.973E+09	24.25	0.0002
C	2.438E+09	1	2.438E+09	8.48	0.0102
AB	3.872E+09	1	3.872E+09	13.46	0.0021
AC	2.913E+09	1	2.913E+09	10.13	0.0058
C^2	1.392E+09	1	1.392E+09	4.84	0.0428
Pure error	2.438E+09	9	2.708E+08		
Cor total	3.311E+10	23			

Final equation (coded factors): $(ee\ \mathbf{3e})^3$ = 763000 + 23146.65A*** + 19682.70B*** – 11638.99C** – 15556.51AB*** + 13949.17AC*** – 30467.95C^2**

**R^2**	0.8555
**Adjusted R^2**	0.8013
**Predicted R^2**	0.6739
**Adeq Precision**	12.3325

Table S6: validation of the model (Design-Expert®). Prediction of the responses at the selected point (A = 13 μM; B = 0.5 mM; C = 50 mM) and related experimental results. CI = confidence interval.

Response	Predicted	Experimental[a]	95% CI
Conversion (%)	17	18	16-19
**3e/2e**	0.85	0.88	0.82 - 0.88
ee (*S*)-**2e** (%)	99	99	99 - 100
ee (*S*)-**3e** (%)	89	89	88 - 90

[a] Mean of duplicate experiments. Data from the models were rounded for clarity.

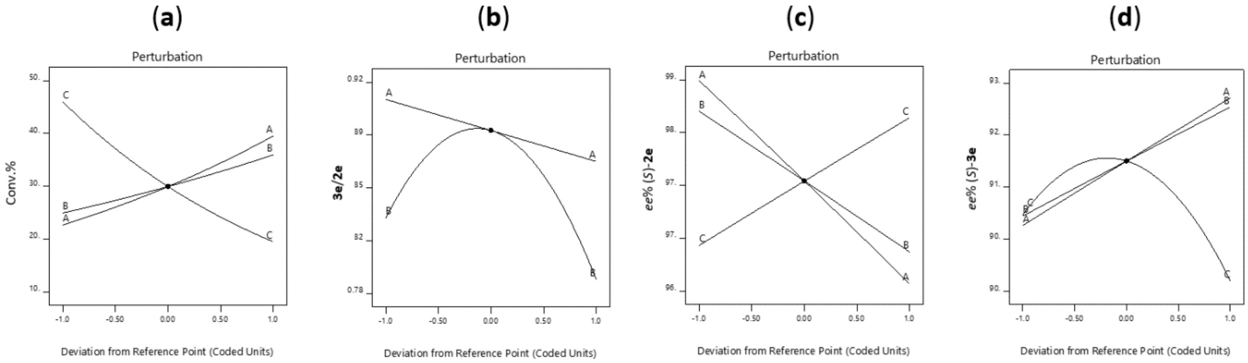

Figure S4: perturbation plots (Design-Expert®) of the four responses: conversion % (**a**), ratio **3e/2e** (**b**), *ee* of (*S*)-**2e** (**c**) and *ee* of (*S*)-**3e** (**d**).

4. HLADH total turnover number

Table S7: HLADH total turnover number (TTN) in disproportionation of **1a-g** at maximum product formation[a]

Entry	Substrate	[Substrate] (mM)	[Total product] (mM)	TTN_{HLADH}
1	**1a**	75	38	2923
2	**1a**	75[b]	59.5	2288
3	**1b**	50	13.5	1038
4	**1c**	10-30	3.6	277
5	**1d**	30	7.5	577
6	**1e**	50	5.5	424
7	**1f**	50	9.5	731
8	**1g**	30	8	615

[a] Conditions: phosphate buffer (50 mM, pH 7.5), HLADH (13 µM), 0.5 mM NAD^+, 30 °C, 24 h.

[b] Second aliquot of fresh enzyme added after 24 h reaction time. Total reaction time 48 h.

5. Synthesis of substrates and reference compounds

Only compounds used as substrates for the biotransformations, reference compounds, and intermediates that were employed during their synthesis are described. 2-(4-methylphenyl)propanal **1e** and 2-(4-methoxyphenyl)propanal **1f** were a kind gift from C. S. Fuchs, prepared according to a previously published procedure.[2]

General procedure for the synthesis of racemic aldehydes 1b-d

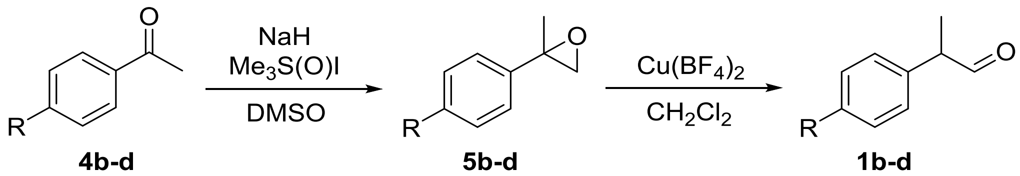

R = F (**b**), CF_3 (**c**), Br (**d**)

Methyloxiranes **5b-d** were synthesized according to the procedure reported by Theodorou and co-workers.[3] To a stirring solution of NaH (60% in mineral oil, 520 mg, 13.00 mmol, 1.3 eq.) in 50 mL of dry DMSO under Ar, $Me_3S(O)I$ (2.862 g, 13.00 mmol, 1.3 eq.) was added at room temperature. The reaction mixture was left stirring for 1 h at room temperature. A solution of aryl methyl ketone **4** (10.00 mmol) in dry DMSO was added dropwise and the reaction mixture was left stirring overnight. The reaction mixture was diluted with H_2O, followed by extractions with EtOAc; the crude product was purified by flash chromatography, eluting with the appropriate mixture of PE/EtOAc.

2-(4-fluorophenyl)-2-methyloxirane (5b): yield 48%; R_f 0.4 (PE/EtOAc 95:5). ^{1}H NMR (300 MHz, $CDCl_3$): δ = 7.38 – 7.28 (m, 2H), 7.11 – 6.94 (m, 2H), 2.97 (d, *J* = 5.3 Hz, 2H), 2.77 (d, *J* = 5.3 Hz, 2H), 1.70 (s, 3H). Spectroscopic data agree with those reported in literature.[3]

2-methyl-2-(4-(trifluoromethyl)phenyl)oxirane (5c): yield 66%; R_f 0.4 (PE/EtOAc 90:10). ^{1}H NMR (300 MHz, $CDCl_3$): δ = 7.60 (d, *J* = 8.1 Hz, 1H), 7.48 (d, *J* = 8.1 Hz, 1H), 3.01 (d, *J* = 5.3 Hz, 1H), 2.77 (d, *J* = 5.2 Hz, 1H), 1.74 (s, 2H). Spectroscopic data correspond to those reported in literature.[3]

2-(4-bromophenyl)-2-methyloxirane (5d): yield 69%; R_f 0.4 (PE/EtOAc 90:10). ^{1}H NMR (300 MHz, $CDCl_3$): δ = 7.49 – 7.42 (m, 2H), 7.30 – 7.18 (m, 2H), 2.98 (d, *J* = 5.4 Hz, 1H), 2.75 (d, *J* = 5.4 Hz, 1H), 1.70 (s, 3H). Spectroscopic data correspond to those reported in literature.[3]

Aldehydes **1b-d** were synthesized starting from the corresponding methyloxiranes, according to a modified procedure based on the work of Robinson and co-workers.[4] To a stirring solution of the methyloxyrane **5** (8.00 mmol) in 40 mL of dry CH_2Cl_2 under Ar, $Cu(BF_4)_2{\cdot}xH_2O$ (20% w/w) was added and the reaction mixture was stirred to completion. The reaction mixture was washed with H_2O and extracted with CH_2Cl_2; the crude product was purified by flash chromatography, eluting with the appropriate mixture of PE/EtOAc, yielding the desired aldehyde **1**.

2-(4-fluorophenyl)propanal (1b): obtained in 64% yield, starting from 2-(4-fluorophenyl)-2-methyloxirane **5b**. R_f 0.4 (PE/EtOAc 90:10). ^{1}H NMR (300 MHz, CDCl3): δ = 9.66 (d, *J* = 1.3 Hz, 1H), 7.23 – 7.00 (m, 4H), 3.63 (q, *J* = 7.0 Hz, 1H), 1.44 (d, *J* = 7.1 Hz, 3H). EI MS (70 eV, m/z (%)): 152.1 (M^+, 24), 123.0 (21), 103.1 (100), 77.1 (28). Spectroscopic data agree with those reported in literature.[3]

2-(4-(trifluoromethyl)phenyl)propanal (1c): obtained in 64% yield, starting from 2-methyl-2-(4-(trifluoromethyl)phenyl)oxirane **5c**. R_f 0.4 (PE/EtOAc 90:10). ^{1}H NMR (300 MHz, CDCl3): δ = 9.70 (d, *J* = 1.0 Hz, 1H), 7.64 (d, *J* = 8.1 Hz, 2H), 7.34 (d, *J* = 8.1 Hz, 2H), 3.73 (q, *J* = 7.1 Hz, 1H), 1.49 (d, *J* = 7.1 Hz, 3H). EI MS (70 eV, m/z (%)): 202.1 (M^+, 9), 183.1 (6), 173.1 (100), 153.1 (23), 133.1 (41). Spectroscopic data correspond to those reported in literature.[5]

2-(4-bromophenyl)propanal (1d): obtained in 72% yield, starting from epoxide 2-(4-bromophenyl)-2-methyloxirane **5d**. R_f 0.5 (PE/EtOAc 95:5). ^{1}H NMR (300 MHz, $CDCl_3$): δ = 9.66 (d, *J* = 1.3 Hz, 1H), 7.51 (d, *J* = 8.4 Hz, 2H), 7.09 (d, *J* = 8.4 Hz, 2H), 3.61 (qd, *J* = 7.0, 0.8 Hz, 1H), 1.43 (d, *J* = 7.1 Hz, 3H). EI MS (70 eV, m/z (%)): 214.0 (M+2, 16), 212.0 (M^+, 16), 185.0 (86), 183.0 (89), 104.1 (100), 77.1 (23). Spectroscopic data correspond to those reported in literature.[3]

Synthesis of reference 2-(4-isobutylphenyl)propan-1-ol (2g) and 2-(4-isobutylphenyl)propanal (1g)

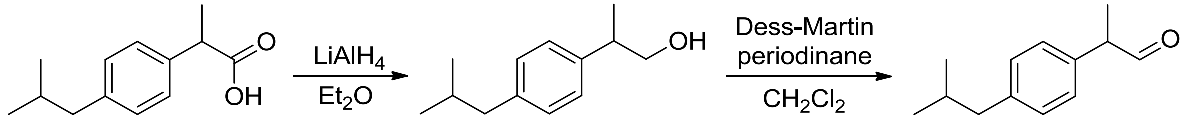

Ibuprofen **3g** (2-(4-isobutylphenyl)propanoic acid, 500 mg, 2.42 mmol) was dissolved in 7 mL of dry EtO_2 under Ar, cooled to 0°C and $LiAlH_4$ (118 mg, 3.12 mmol, 1.3 eq.) was added. After completion, the excess of hydride was cautiously decomposed by adding a solution of NaOH 0.75M until the formation of a white precipitate occurred. The precipitate was removed by filtration under reduced pressure, the solution was concentrated, and the crude was purified by flash chromatography (PE/EtOAc 80:20), affording the desired alcohol **2g** in 99% yield (463 mg, 2.41 mmol).

To a stirring solution of alcohol **2g** (200 mg, 1.04 mmol) in 13 mL of dry CH_2Cl_2 under Ar, Dess-Martin periodinane (573 mg, 1.35 mmol, 1.3 eq.) was added and the reaction mixture was stirred at room temperature for 1 hour. The mixture was then diluted with CH_2Cl_2, washed with a saturated solution of $NaHCO_3$ and extracted with CH_2Cl_2. After concentration, the crude was purified by flash chromatography (PE/EtOAc 98:2), yielding 147 mg of the aldehyde **1g** (0.77 mmol, 74% yield).

2-(4-isobutylphenyl)propan-1-ol (2g): R_f 0.4 (PE/EtOAc 80:20). ^{1}H NMR (300 MHz, $CDCl_3$): δ = 7.19 – 7.04 (m, 4H), 3.68 (d, *J* = 6.8 Hz, 2H), 2.92 (dq, *J* = 6.9, 6.9 Hz, 1H), 2.45 (d, *J* = 7.2 Hz, 2H), 1.86 (m, 1H), 1.48 (bs, 1H), 1.27 (d, *J* = 7.0 Hz, 3H), 0.91 (d, *J* = 6.6 Hz, 6H). EI MS (70 eV, m/z (%)): 192.0 (M^+, 15), 161.0 (100), 119.1 (26), 105.1 (8), 91.1 (13). Spectroscopic data agree with those reported in literature.[5]

2-(4-isobutylphenyl)propanal (1g): R_f 0.5 (PE/EtOAc 90:10). ^{1}H NMR (300 MHz, $CDCl_3$): δ = 9.67 (d, *J* = 1.5 Hz, 1H), 7.19 – 7.08 (m, 4H), 3.60 (qd, *J* = 7.0, 1.3 Hz, 1H), 2.47 (d, *J* = 7.2 Hz, 2H), 1.93 - 1.77 (m, 1H), 1.43 (d, *J* = 7.1 Hz, 3H), 0.90 (d, *J* = 6.6 Hz, 6H). EI MS (70 eV, m/z (%)): 190.0 (M^+, 8), 161.0 (100), 119.1 (26). Spectroscopic data correspond with those reported in literature.[5]

General procedure for the synthesis of racemic alcohols 2b-f

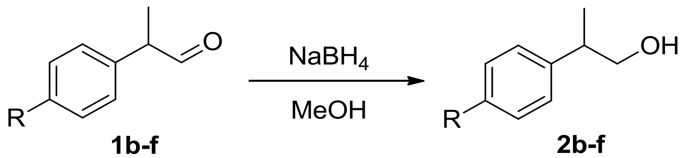

R = F (**b**), CF_3 (**c**), Br (**d**),CH_3 (**e**), CH_3O (**f**)

To a stirring solution of the aldehyde **1** (1.50 mmol) in 7 mL of MeOH, under Ar and cooled to 0°C, $NaBH_4$ (2.25 mmol, 1.5 eq.) was added. After completion. the reaction was quenched with HCl 2N and extracted with EtOAc. After concentration, the crude was chromatographed (PE/EtOAc) to give the pure desired alcohol **2**.

2-(4-fluorophenyl)propan-1-ol (2b): obtained in 60% yield, starting from 2-(4-fluorophenyl)propanal **1b**. R_f 0.4 (PE/EtOAc 70:30). ^{1}H NMR (300 MHz, $CDCl_3$): δ = 7.20 (dd, *J* = 8.6, 5.5 Hz, 2H), 7.01 (t, *J* = 8.7 Hz, 2H), 3.67 (d, *J*

= 7.0 Hz, 2H), 2.94 (dq, *J* = 6.9, 6.9 Hz, 1H), 1.37 (bs, 1H), 1.26 (d, *J* = 7.0 Hz, 3H). EI MS (70 eV, m/z (%)): 168.1 (M^+, 27), 123.0 (100), 104.1 (38), 77.1 (10). Spectroscopic data agree to those reported in literature.[6]

2-(4-(trifluoromethyl)phenyl)propan-1-ol (2c): obtained in 75% yield, starting from 2-(4-isobutylphenyl)propanal **1c**. R_f 0.4 (PE/EtOAc 80:20). ^{1}H NMR (300 MHz, $CDCl_3$): δ = 7.59 (d, *J* = 8.1 Hz, 2H), 7.36 (d, *J* = 8.2 Hz, 2H), 3.74 (d, *J* = 6.7 Hz, 2H), 3.03 (dq, *J* = 6.9, 6.9 Hz, 1H), 1.40 (bs, 1H) 1.30 (d, *J* = 7.0 Hz, 3H). EI MS (70 eV, m/z (%)): 204.1 (M^+, 10), 185.1 (11), 173.1 (100), 154.1 (47), 133.1 (53), 105.1 (33).Spectroscopic data correspond to those reported in literature.[5]

2-(4-bromophenyl)propan-1-ol (2d): obtained in 78% yield from 2-(4-bromophenyl)propanal **1d**. R_f 0.4 (PE/EtOAc 70:30). ^{1}H NMR (300 MHz, $CDCl_3$): δ = 7.45 (d, *J* = 8.4 Hz, 2H), 7.12 (d, *J* = 8.4 Hz, 2H), 3.67 (d, *J* = 7.1 Hz, 2H), 2.91 (dq, *J* = 6.9, 6.9 Hz, 1H), 1.40 (bs, 1H), 1.25 (d, *J* = 7.0 Hz, 3H). EI MS (70 eV, m/z (%)): 216.0 (M+2, 22), 214.0 (M^+, 23), 185.1 (93), 183.1 (98), 104.1 (100), 77.1 (22). Spectroscopic correspond to those reported in literature.[7]

2-(4-methylphenyl)propan-1-ol (2e): obtained in 90% yield, starting from 2-(4-methylphenyl)propanal **1e**.[2] R_f 0.5 (PE/EtOAc 70:30). ^{1}H NMR (300 MHz, $CDCl_3$): δ =7.13 (s, 4H), 3.67 (d, *J* = 6.4 Hz, 2H), 2.91 (dq, *J* = 6.9, 6.9 Hz, 1H), 2.33 (s, 3H), 1.38 (bs, 1H), 1.25 (d, *J* = 7.0 Hz, 3H). EI MS (70 eV, m/z (%)): 150.1 (M^+, 19), 119.1 (100), 91.1 (20), 77.1 (6). Spectroscopic data correspond to those reported in literature.[8]

2-(4-methoxyphenyl)propan-1-ol (2f): obtained in 88% yield, starting from 2-(4-methoxyphenyl)propanal **1f**.[2] R_f 0.4 (PE/EtOAc 70:30). ^{1}H NMR (300 MHz, $CDCl_3$): δ = 7.17 (d, *J* = 8.6 Hz, 2H), 6.88 (d, *J* = 8.7 Hz, 2H), 3.80 (s, 3H), 3.66 (d, *J* = 6.4 Hz, 2H), 2.91 (dq, *J* = 7.0, 6.9 Hz, 1H), 1.30 (s, 1H), 1.25 (d, *J* = 7.0 Hz, 3H). EI MS (70 eV, m/z (%)): 1.1 (M^+, 14), 135.1 (100), 105.1 (14), 91.1 (10), 77.1 (7). Spectroscopic data agree to those reported in literature.[7]

General procedure for the synthesis of racemic acids 3b-f

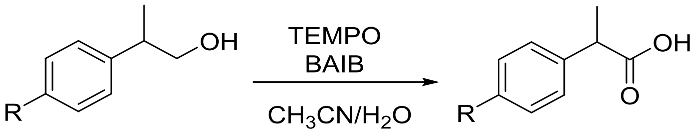

2b-f **3b-f**

R = F (**b**), CF_3 (**c**), Br (**d**), CH_3 (**e**), CH_3O (**f**)

To a stirring solution of the alcohol **2** (0.5 mmol) in 3 mL of a mixture 1:1 of CH_3CN/H_2O, TEMPO (2,2,6,6-tetramethyl-1-piperidinyloxy, free radical, 15 mg, 0.1 mmol, 0.2 eq.) and (diacetoxyiodo)-benzene (BAIB, 354 mg, 1.1 mmol, 2.2 eq.) were added. The reaction was stirred overnight at room temperature. After completion, the mixture was quenched with a solution $Na_2S_2O_3$ 10 % and extracted with EtOAc. The organic phase was extracted with a saturated solution of $NaHCO_3$; the combined aqueous fractions were acidified with HCl 3N and extracted with EtOAc. The solvent was evaporated under reduced pressure, affording the desired carboxylic acid **3**.

2-(4-fluorophenyl)propanoic acid (3b): obtained in 45% yield, starting from 2-(4-fluorophenyl)propan-1-ol (**2b**). R_f 0.2 (PE/EtOAc 70:30). ^{1}H NMR (300 MHz, $CDCl_3$): δ = 7.28 (dd, *J* = 8.6, 5.3 Hz, 2H), 7.01 (t, *J* = 8.70 Hz, 2H), 3.74 (q, *J* = 7.2 Hz, 1H), 1.51 (d, *J* = 7.2 Hz, 3H). EI MS (70 eV, m/z (%)): 154.1 (M^+, 16), 123.1 (100), 103.1 (41), 77.1 (9). Spectroscopic data correspond to those reported in literature.[9]

2-(4-(trifluoromethyl)phenyl)propanoic acid (3c): obtained in 46% yield, starting from 2-(4-(trifluoromethyl)phenyl)propan-1-ol (**2c**). R_f 0.2 (PE/EtOAc 70:30). ^{1}H NMR (300 MHz, $CDCl_3$): δ = 10.16 (bs, 1H), 7.59 (d, *J* = 8.2 Hz, 2H), 7.44 (d, *J* = 8.2 Hz, 2H), 3.81 (q, *J* = 7.2 Hz, 1H), 1.54 (d, *J* = 7.2 Hz, 3H). EI MS (70 eV, m/z (%)): 218.1 (M^+, 30), 199.1 (11), 173.1 (100), 153.1 (24), 133.1 (35), 105.1 (12), 77.1 (5). Spectroscopic data agree to those reported in literature.[10]

2-(4-bromophenyl)propanoic acid (3d): obtained in 66% yield, starting from 2-(4-bromophenyl)propan-1-ol (**2d**). R_f 0.2 (PE/EtOAc 70:30). ^{1}H NMR (300 MHz, $CDCl_3$): δ = 7.46 (d, *J* = 8.5 Hz, 2H), 7.20 (d, *J* = 8.4 Hz, 2H), 3.70 (q, *J* = 7.2 Hz, 1H), 1.50 (d, *J* = 7.2 Hz, 3H). EI MS (70 eV, m/z (%)): 230.0 (M+2, 32), 228.0 (M^+, 33), 185.1 (96), 183.1 (100), 104.1 (95), 77.1 (27). Spectroscopic data are consistent with those reported in literature.[11]

2-(4-methylphenyl)propanoic acid (3e): obtained in 55% yield, starting from 2-(4-methylphenyl)propan-1-ol (**2e**). R_f 0.2 (PE/EtOAc 70:30). ^{1}H NMR (300 MHz, $CDCl_3$): δ = 7.21 (d, *J* = 8.1 Hz, 2H), 7.13 (d, *J* = 8.0 Hz, 2H), 3.70 (q, *J* = 7.2 Hz, 1H), 2.33 (d, *J* = 4.3 Hz, 3H), 1.49 (d, *J* = 7.2 Hz, 3H). EI MS (70 eV, m/z (%)): 164.1 (M^+, 27), 119.1 (100), 91.1 (18). Spectroscopic data agree to those reported in literature.[9]

2-(4-methoxyphenyl)propanoic acid (3f): obtained in 44% yield, starting from 2-(4-methoxyphenyl)propan-1-ol (**2f**). R_f 0.2 (PE/EtOAc 70:30). ^{1}H NMR (300 MHz, $CDCl_3$): δ = 7.24 (d, *J* = 8.8 Hz, 2H), 6.86 (d, *J* = 8.7 Hz, 2H), 3.79 (s, 3H), 3.68 (q, *J* = 7.2 Hz, 1H), 1.48 (d, *J* = 7.2 Hz, 3H). EI MS (70 eV, m/z (%)): 180.1 (M^+, 26), 135.1 (100), 105.1 (13), 77.1 (8).Spectroscopic agree to those reported in literature.[9]

<u>**Determination of the absolute configuration**</u>

Enantioenriched reference compounds, when not commercially available, were synthesized by the following general procedures:

Typical enzymatic protocol for the synthesis of reference (*S*)-2b-g

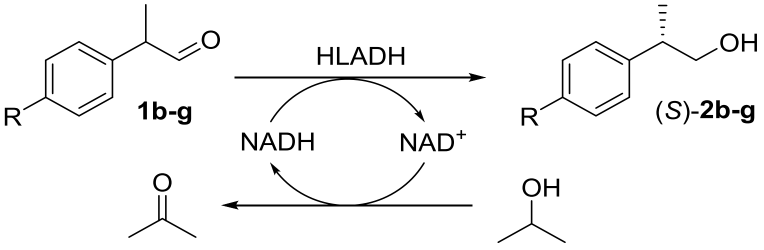

R = F (**b**), CF_3 (**c**), Br (**d**), CH_3 (**e**), CH_3O (**f**), ^{i}Bu (**g**)

Compounds (*S*)-**2b-d** were synthesized according to a modified procedure based on the work of Galletti and co-workers.[12] Reactions were run in more replicates. An aliquot of lyophilized whole cells of *E. coli* containing HLADH enzyme (5 mg) was added to a phosphate buffer solution (final volume 1 mL, 50 mM, pH 7.5) containing the aldehyde **1** (C_{fin} = 30 mM), the co-substrate (isopropanol, 25 µL, 325 mM) and the nicotinamide cofactor (NAD^+, 0.33 mg, C_{fin} = 0.5 mM). The reaction mixture was incubated at 30 °C, 120 rpm for 24 h. Then the reaction mixture from the replicates was combined and extracted with EtOAc; the combined organic fractions were dried over Na_2SO_4 and the solvent removed under vacuum. The crude was purified by flash chromatography with the appropriate mixture of PE/EtOAc to afford the desired product (*S*)-**2**.

(***S***)**-2-(4-fluorophenyl)propan-1-ol ((*S*)-2b)**: $[\alpha]^{20}_{D}$ = -11.2 (c = 1.0, $CHCl_3$); lit. value reported for (*R*)-enantiomer: $[\alpha]^{24}_{D}$ = +16.2 (c = 1.0, $CHCl_3$).[13]

(***S***)**-2-(4-(trifluoromethyl)phenyl)propan-1-ol ((*S*)-2c)**: $[\alpha]^{20}_{D}$ = -11.9 (c = 1.0, $CHCl_3$); lit. value: $[\alpha]^{22}_{D}$ = -15.2 (c = 0.51, $CHCl_3$).[5]

(***S***)**-2-(4-bromophenyl)propan-1-ol ((*S*)-2d)**: $[\alpha]^{20}_{D}$ = -11.4 (c = 1.0, $CHCl_3$); lit. value reported for (*R*)-enantiomer: $[\alpha]^{20}_{D}$ = +8.6 (c = 1.0, $CHCl_3$).[14]

(***S***)**-2-(4-methylphenyl)propan-1-ol ((*S*)-2e)**: $[\alpha]^{20}_{D}$ = -15.4 (c = 1.0, $CHCl_3$); lit. value: $[\alpha]^{20}_{D}$ = -19.2 (c = 0.82, $CHCl_3$).[15]

(***S***)**-2-(4-methoxyphenyl)propan-1-ol ((*S*)-2f)**: $[\alpha]^{20}_{D}$ = -11.8 (c = 1.0, $CHCl_3$); lit. value: $[\alpha]^{21}_{D}$ = -13.1 (c = 2.1, $CHCl_3$).[16]

(***S***)**-2-(4-isobutylphenyl)propan-1-ol ((*S*)-2g)**: $[\alpha]^{20}_{D}$ = -13.6 (c = 1.0, $CHCl_3$); lit. value: $[\alpha]^{22}_{D}$ = -14.6 (c = 1.0, $CHCl_3$).[5]

General oxidation protocol for the synthesis of reference (*S*)-3b-f

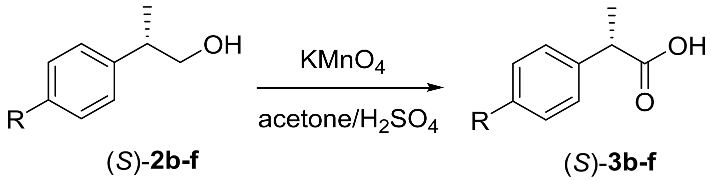

R = F (**b**), CF_3 (**c**), Br (**d**), CH_3 (**e**), CH_3O (**f**)

Compounds (*S*)-**3b-d** were synthesized according to the procedure reported by Galletti and co-workers.[12] No racemization occurred during the reaction and the absolute configuration of the chiral center was conserved. To a stirring solution of the (*S*)-alcohol ((*S*)-**2**) (0.2 mmol) in 3 mL of a mixture of acetone and H_2SO_4 3N (1:1) solid $KMnO_4$ (125 mg, 0.8 mmol, 4eq.) was added and the reaction was stirred at 0 °C for 4 hours. After completion, the mixture was diluted with HCl 1N and solid Na_2SO_3 was added until the discoloration of the solution. Then the aqueous phase was extracted with EtOAc; the organic fractions were then extracted with a 2% NaOH solution. The basic solution was acidified to pH = 2 with HCl 3N and extracted with EtOAc; the collected organic fractions were dried over Na_2SO_4 and the solvent was evaporated, leading to the final product (*S*)-**3**.

(***S***)**-2-(4-fluorophenyl)propanoic acid ((*S*)-3b)**: $[\alpha]^{20}_{D}$ = +52.6 (c = 1.0, MeOH); lit. value: $[\alpha]_{D}$ = +70 (c = 1.0, MeOH).[17]

(***S***)**-2-(4-(trifluoromethyl)phenyl)propanoic acid ((*S*)-3c)**: $[\alpha]^{20}_{D}$ = +32.6 (c = 1.0, EtOH).

(***S***)**-2-(4-bromophenyl)propanoic acid ((*S*)-3d)**: $[\alpha]^{20}_{D}$ = +26.1 (c = 1.0, MeOH); lit. value: $[\alpha]^{22}_{D}$ = +46.9 (c = 1.5, MeOH).[18]

(***S***)**-2-(4-methylphenyl)propanoic acid ((*S*)-3e)**: $[\alpha]^{20}_{D}$ = +10.2 (c = 1.0, $CHCl_3$); lit. value: $[\alpha]^{21}_{D}$ = +46.8 (c = 1.3, $CHCl_3$).[19]

(*S*)-2-(4-methoxyphenyl)propanoic acid ((*S*)-3f): $[\alpha]^{20}_D$ = +49.3 (c = 1.0, EtOH); lit. value: $[\alpha]_D$ = +69.1 (c = 2.0, EtOH).[18]

6. Chiral GC analyses

GC analyses were carried out on an Agilent 7890A, with FID detector, using H_2 as carrier gas; flow 1 mL/min, detector temperature 250 °C, injector temperature 250 °C, split ratio 50:1. Chiral columns were employed for the determination of both conversions and enantiomeric excesses.

Analytical data for 2-phenylpropanal (1a) purchased

Column: Macherey-Nagel Hydrodex β-TBDAc (25 m x 0.25 mm x 0.25 μm)
Temperature program: 110 °C, hold 10 min; 2 °C/min to 123 °C, hold 1.5 min; 10 °C/min to 200 °C, hold 1 min.
Retention times: t_R (1) = 8.2 min, t_R (2) = 8.8 min

Analytical data for 2-phenylpropan-1-ol (2a)

Column: Macherey-Nagel Hydrodex β-TBDAc (25 m x 0.25 mm x 0.25 μm)
Temperature program: 110 °C, hold 10 min; 2 °C/min to 123 °C, hold 1.5 min; 10 °C/min to 200 °C, hold 1 min.
Retention times: t_R (*R*) = 14.1 min, t_R (*S*) = 15.4 min

Reference *rac*-2-phenylpropan-1-ol (**2a**) purchased

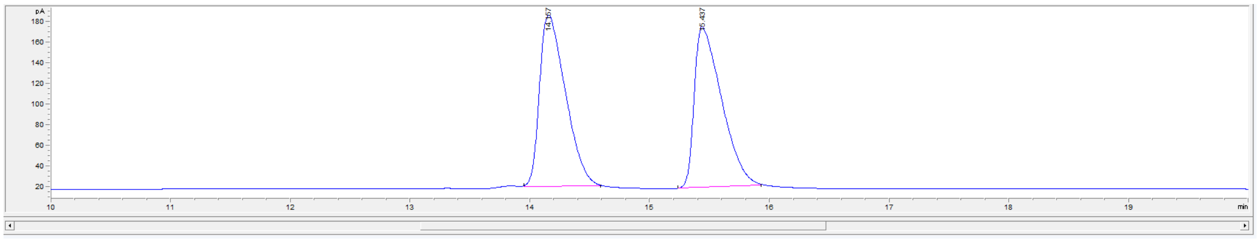

Reference (*R*)-2-phenylpropan-1-ol ((*R*)-**2a)** purchased

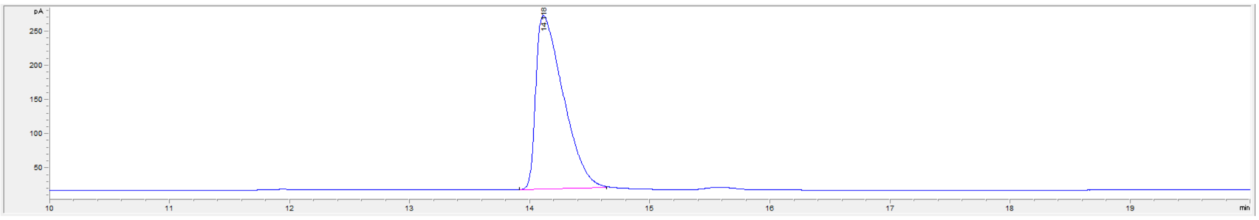

(*S*)-2-phenylpropan-1-ol ((*S*)-**2a**) from biotransformation

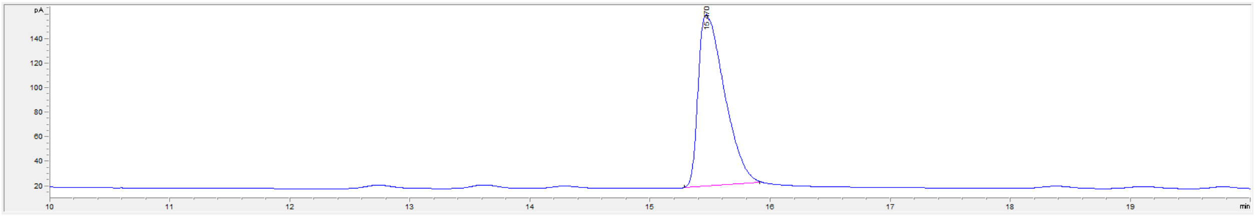

Analytical data for 2-phenylpropanoic acid (3a)

Column: Agilent CP-Chirasil-DEX CB column (25 m x 0.32 mm x 0.25 µm)
Temperature program: 100 °C, hold 5 min; 5 °C/min to 155 °C, hold 8 min, 10 °C/min to 180 °C, hold 1 min.
Retention times: t_R (*S*) = 19.1 min, t_R (*R*) = 19.6 min

Reference *rac*-2-phenylpropanoic acid (**3a**) purchased

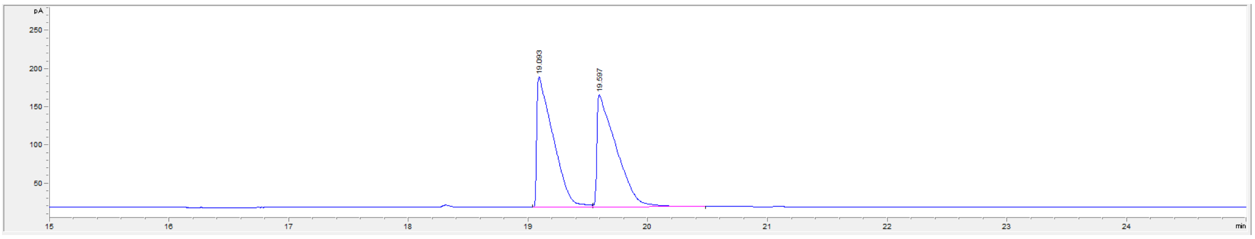

Reference (*S*)-2-phenylpropanoic acid ((*S*)-**3a**) purchased

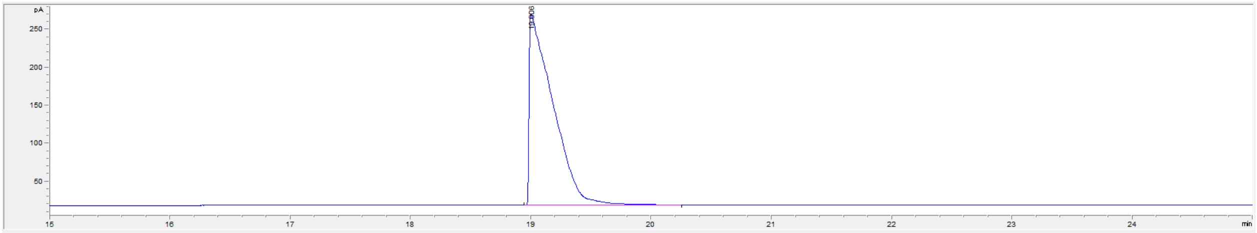

(*S*)-2-phenylpropanoic acid ((*S*)-**3a**) from biotransformation

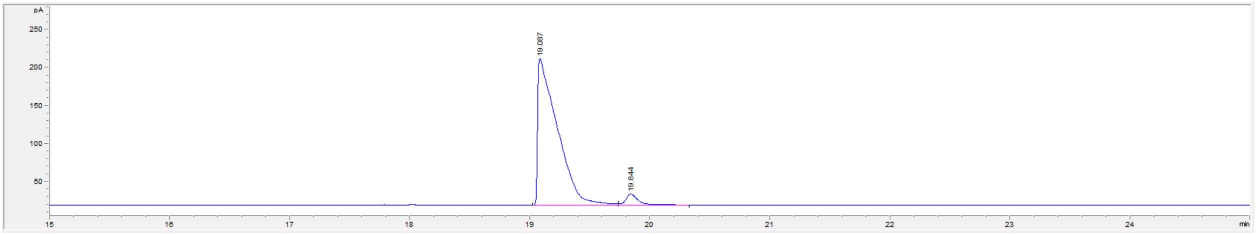

Analytical data for 2-(4-fluorophenyl)propanal (1b)

Column: Macherey-Nagel Hydrodex β-TBDAc (25 m x 0.25 mm x 0.25 µm)
Temperature program: 100 °C, hold 0 min; 10 °C/min to 140 °C, hold 10 min; 10 °C/min to 200 °C, hold 1 min.
Retention times: t_R (1) = 6.3 min, t_R (2) = 6.6 min

Analytical data for 2-(4-fluorophenyl)propan-1-ol (2b)

Column: Macherey-Nagel Hydrodex β-TBDAc (25 m x 0.25 mm x 0.25 µm)
Temperature program: 100 °C, hold 0 min; 10 °C/min to 140 °C, hold 10 min; 10 °C/min to 200 °C, hold 1 min.
Retention times: t_R (*R*) = 8.2 min, t_R (*S*) = 8.8 min

Reference *rac*-2-(4-fluorophenyl)propan-1-ol (**2b**)

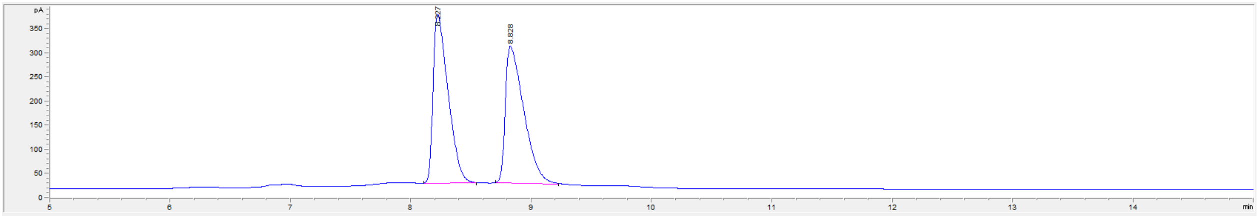

Reference (*S*)-2-(4-fluorophenyl)propan-1-ol ((*S*)-**2b**)

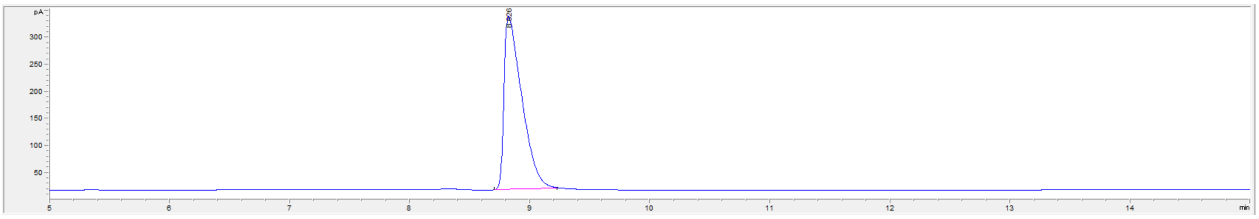

(*S*)-2-(4-fluorophenyl)propan-1-ol ((*S*)-**2b**) from biotransformation

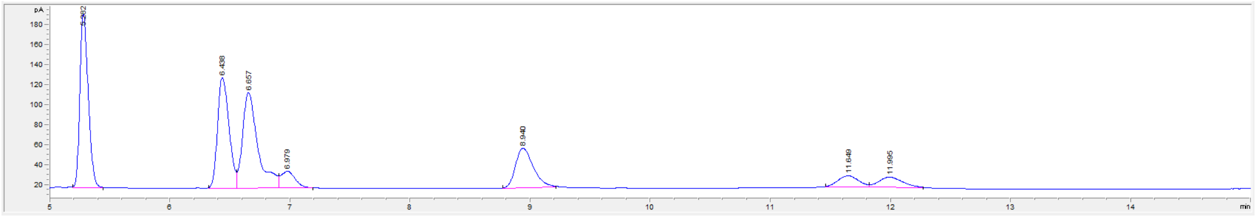

Analytical data for 2-(4-fluorophenyl)propanoic acid (3b)

Column: Agilent CP-Chirasil-DEX CB column (25 m x 0.32 mm x 0.25 µm)
Temperature program: 100 °C, hold 1 min; 5 °C/min to 140 °C, hold 3 min;10 °C/min to 180 °C, hold 5 min.
Retention times: t_R (*S*) =15.9 min, t_R (*R*) = 16.4 min

Reference *rac*-2-(4-fluorophenyl)propanoic acid (**3b**)

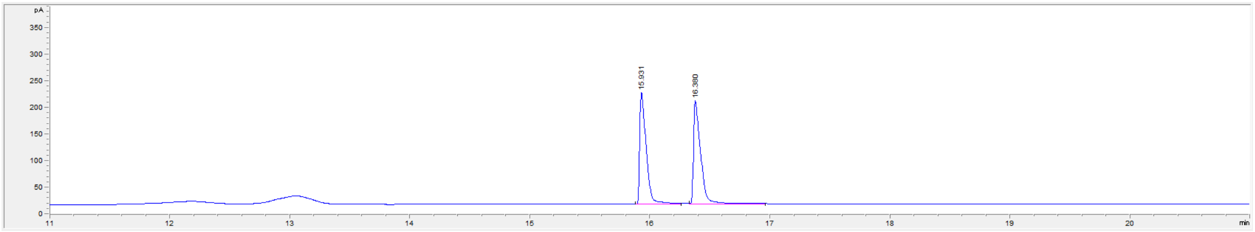

Reference (*S*)-2-(4-fluorophenyl)propanoic acid ((*S*)-3**b**)

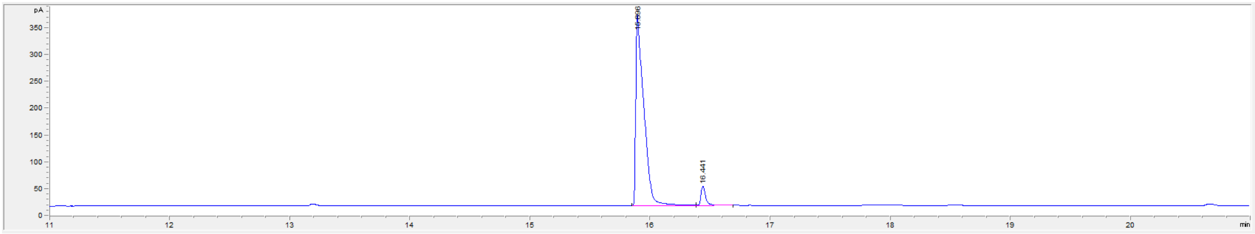

(*S*)-2-(4-fluorophenyl)propanoic acid ((*S*)-**3b**) from biotransformation

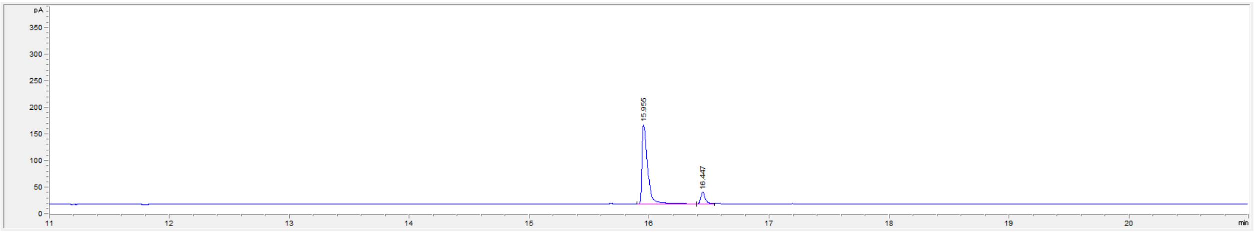

Analytical data for 2-(4-(trifluoromethyl)phenyl)propanal (1c)

Column: Macherey-Nagel Hydrodex β-TBDAc (25 m x 0.25 mm x 0.25 μm)
Temperature program: 100 °C, hold 0 min; 10 °C/min to 140 °C, hold 10 min; 2 °C/min to 150 °C, hold 0 min; 10 °C/min to 200 °C, hold 1 min.
Retention times: t_R (1) = 7.6 min, t_R (2) = 8.0 min

Analytical data for 2-(4-(trifluoromethyl)phenyl)propan-1-ol (2c)

Column: Macherey-Nagel Hydrodex β-TBDAc (25 m x 0.25 mm x 0.25 μm)
Temperature program: 100 °C, hold 0 min; 10 °C/min to 140 °C, hold 10 min; 2 °C/min to 150 °C, hold 0 min; 10 °C/min to 200 °C, hold 1 min.
Retention times: t_R (*R*) = 10.0 min, t_R (*S*) = 10.9 min

Reference *rac*-2-(4-(trifluoromethyl)phenyl)propan-1-ol (**2c**)

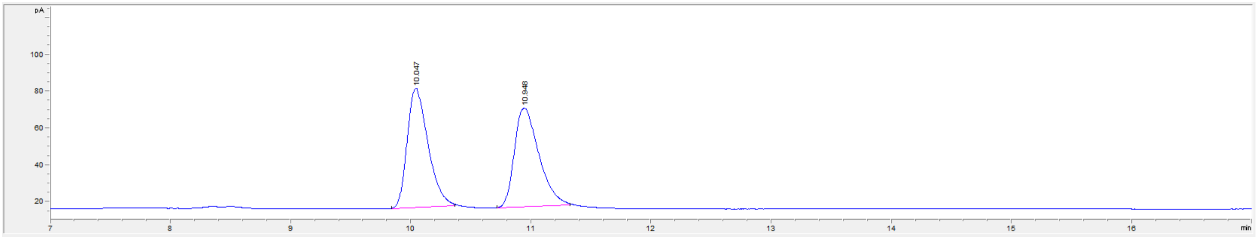

Reference (*S*)-2-(4-(trifluoromethyl)phenyl)propan-1-ol ((*S*)-**2c**)

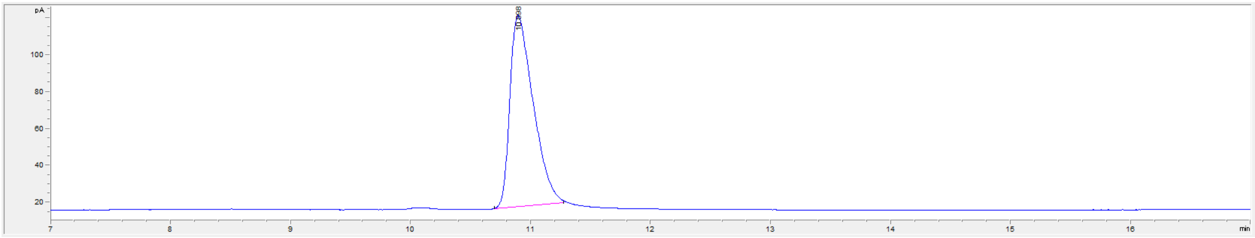

(*S*)-2-(4-(trifluoromethyl)phenyl)propan-1-ol ((*S*)-**2c**)) from biotransformation

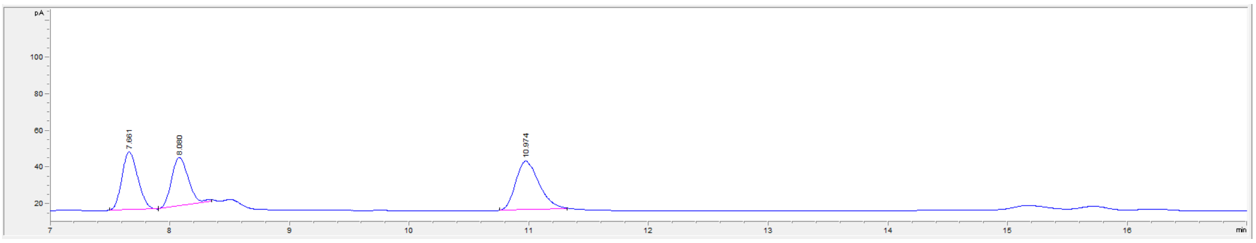

Analytical data for 2-(4-(trifluoromethyl)phenyl)propanoic acid (3c)

Column: Agilent CP-Chirasil-DEX CB column (25 m x 0.32 mm x 0.25 µm)
Temperature program: 100 °C, hold 1 min;5 °C/min to 140 °C, hold 3 min; 10 °C/min to 180 °C, hold 5 min.
Retention times: t_R (*S*) = 16.5 min, t_R (*R*) = 17.2 min

Reference *rac*-2-(4-(trifluoromethyl)phenyl)propanoic acid (**3c**)

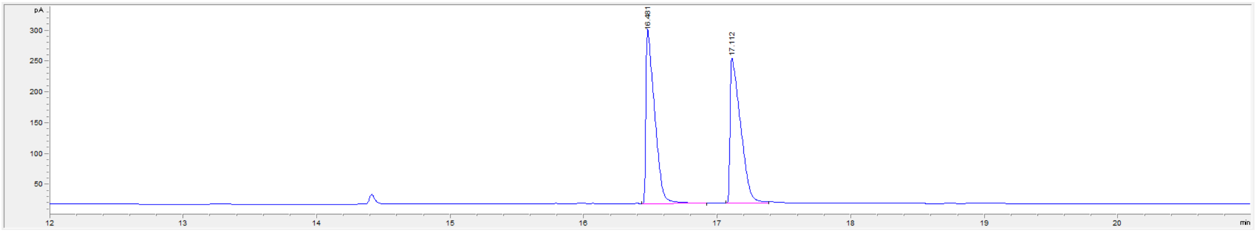

Reference (*S*)-2-(4-(trifluoromethyl)phenyl)propanoic acid ((*S*)-**3c**)

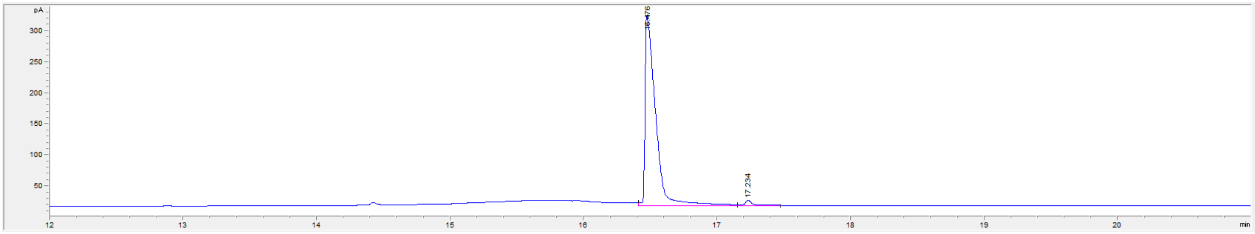

(*S*)-2-(4-(trifluoromethyl)phenyl)propanoic acid ((*S*)-**3c**) from biotransformation

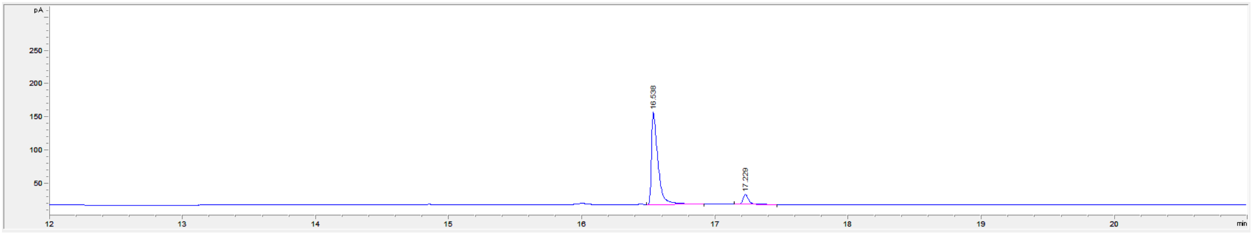

Analytical data for 2-(4-bromophenyl)propanal (1d)

Column: Macherey-Nagel Hydrodex β-TBDAc (25 m x 0.25 mm x 0.25 µm)
Temperature program: 110 °C, hold 1 min; 2 °C/min to 140 °C, hold 15 min; 10 °C/min to 200 °C, hold 1 min.
Retention times: t_R (1) = 26.2 min, t_R (2) = 27.7 min

Analytical data for 2-(4-bromophenyl)propan-1-ol (2d)

Column: Macherey-Nagel Hydrodex β-TBDAc (25 m x 0.25 mm x 0.25 µm)
Temperature program: 110 °C, hold 1 min; 2 °C/min to 140 °C, hold 15 min; 10 °C/min to 200 °C, hold 1 min.
Retention times: t_R (*R*) = 34.2 min, t_R (*S*) = 34.9 min

Reference *rac*-2-(4-bromophenyl)propan-1-ol (**2d**)

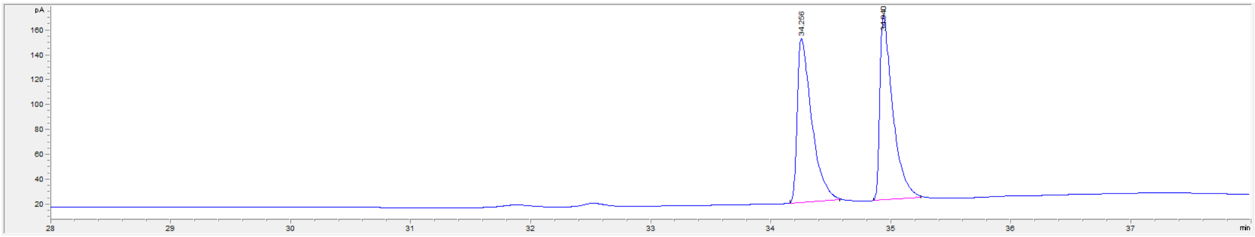

Reference (*S*)-2-(4-bromophenyl)propan-1-ol ((*S*)-**2d**)

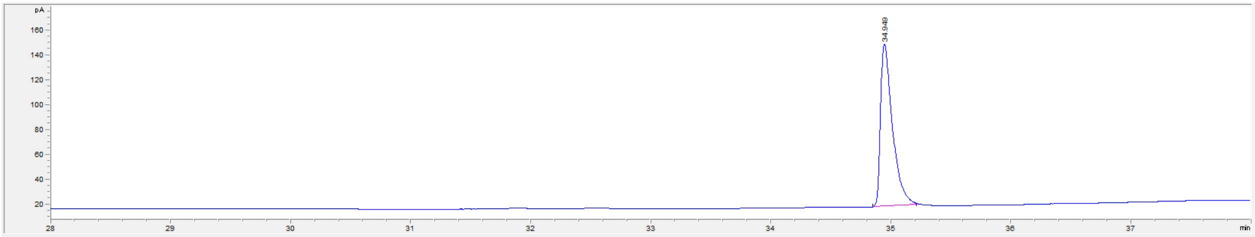

(*S*)-2-(4-bromophenyl)propan-1-ol ((*S*)-**2d**) from biotransformation

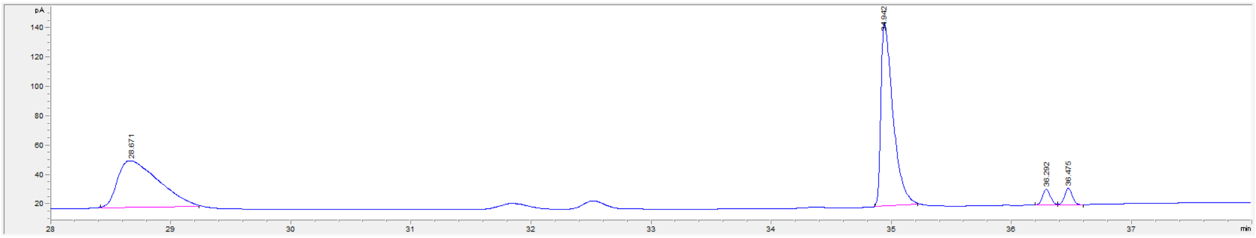

Analytical data for 2-(4-bromophenyl)propanoic acid (3d)

Column: Agilent CP-Chirasil-DEX CB column (25 m x 0.32 mm x 0.25 µm)
Temperature program: 100 °C, hold 1 min; 5 °C/min to 140 °C, hold 3 min; 10 °C/min to 180 °C, hold 13 min.
Retention times: t_R (*S*) = 22.1 min, t_R (*R*) = 23.3 min

Reference *rac*-2-(4-bromophenyl)propanoic acid (**3d**)

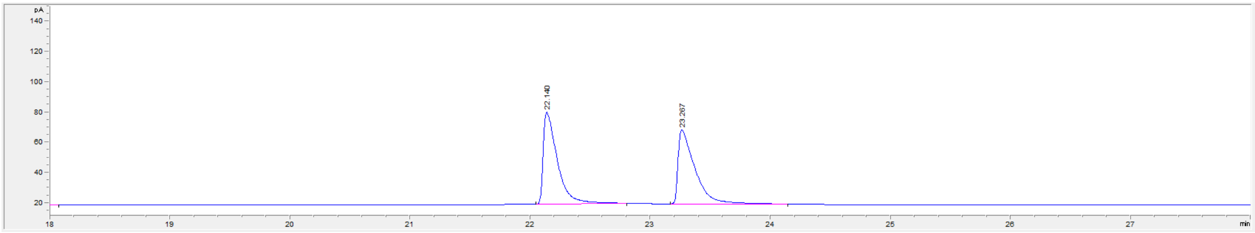

Reference (*S*)-2-(4-bromophenyl)propanoic acid ((*S*)-**3d**)

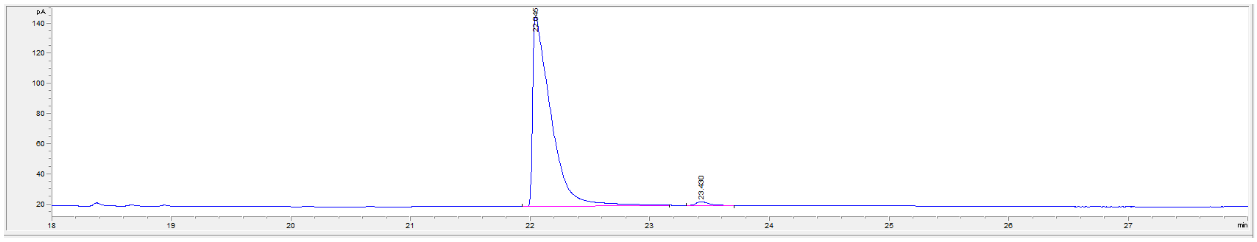

(*S*)-2-(4-bromophenyl)propanoic acid ((*S*)-**3d**) from biotransformation

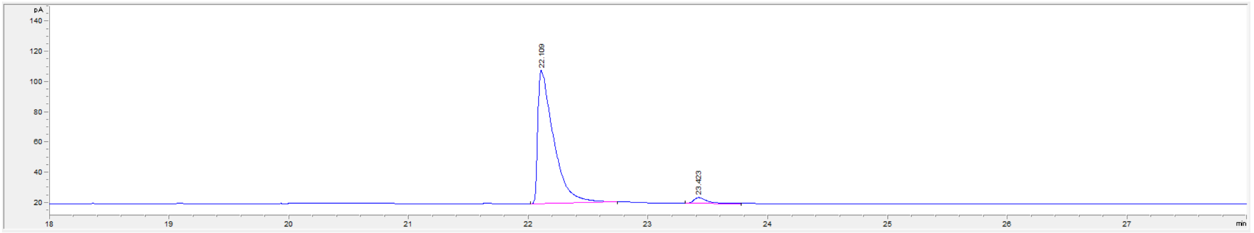

Analytical data for 2-(4-methylphenyl)propanal (1e)

Column: RESTEK Rt-βDEXsm (30 m x 0.32 mm x 0.25 µm)
Temperature program: 100 °C, hold 5 min; 3 °C/min to 130 °C, hold 5 min; 5 °C/min to 140 °C, hold 0 min; 10 °C/min to 220 °C, hold 1 min.
Retention times: t_R (1) = 19.6 min, t_R (2) = 20.0 min

Analytical data for 2-(4-methylphenyl)propan-1-ol (2e)

Column: RESTEK Rt-βDEXsm (30 m x 0.32 mm x 0.25 µm)
Temperature program: 100 °C, hold 5 min; 3 °C/min to 130 °C, hold 5 min; 5 °C/min to 140 °C, hold 0 min; 10 °C/min to 220 °C, hold 1 min.
Retention times: t_R (*R*) = 21.5 min, t_R (*S*) = 21.8 min

Reference *rac*-2-(4-methylphenyl)propan-1-ol (**2e**)

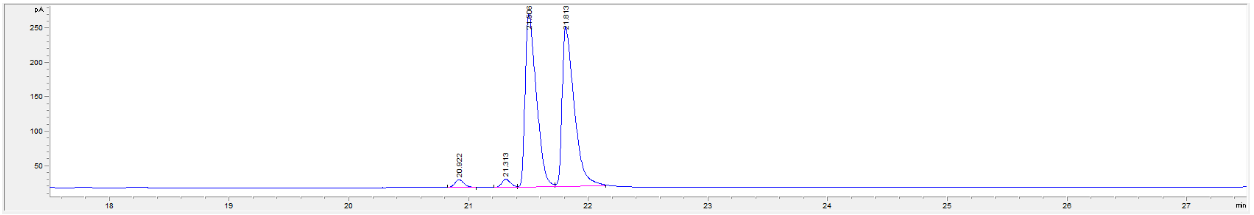

Reference (*S*)-2-(4-methylphenyl)propan-1-ol ((*S*)-**2e**)

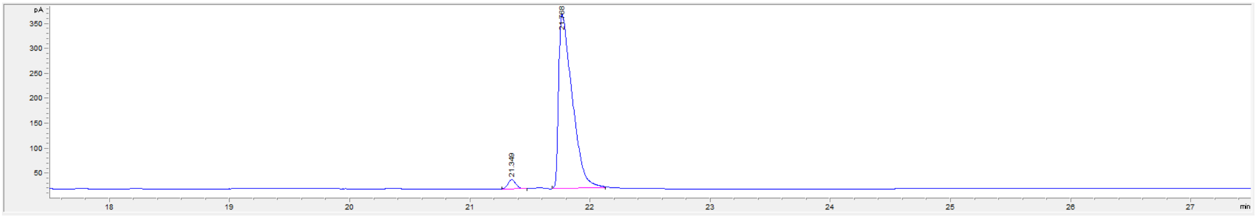

(*S*)-2-(4-methylphenyl)propan-1-ol ((*S*)-**2e**) from biotransformation

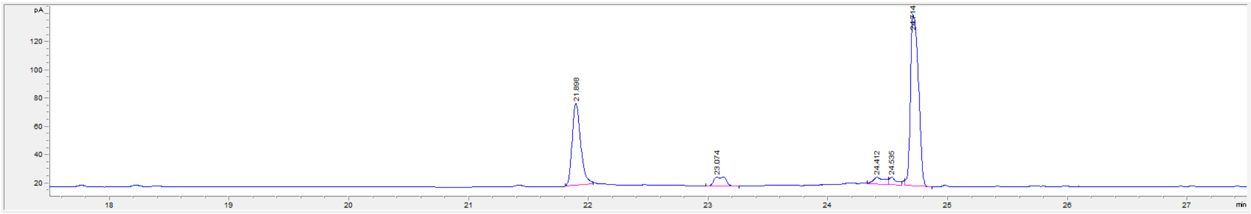

Analytical data for 2-(4-methylphenyl)propanoic acid (3e)

Column: Agilent CP-Chirasil-DEX CB column (25 m x 0.32 mm x 0.25 µm)
Temperature program:
Retention times: t_R (*S*) = 20.1 min, t_R (*R*) = 20.5 min

Reference *rac*-2-(4-methylphenyl)propanoic acid (**3e**)

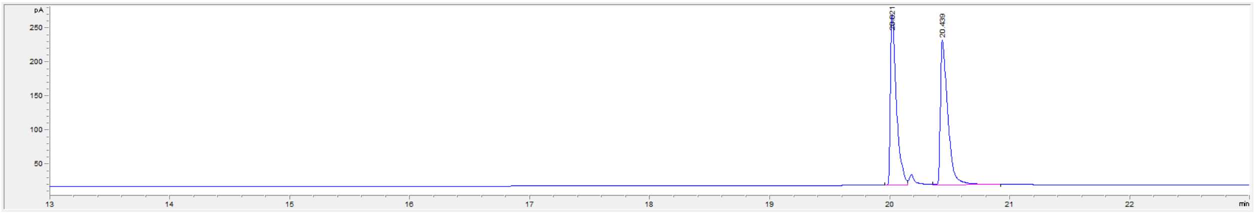

Reference (*S*)-2-(4-methylphenyl)propanoic acid ((*S*)-**3e**)

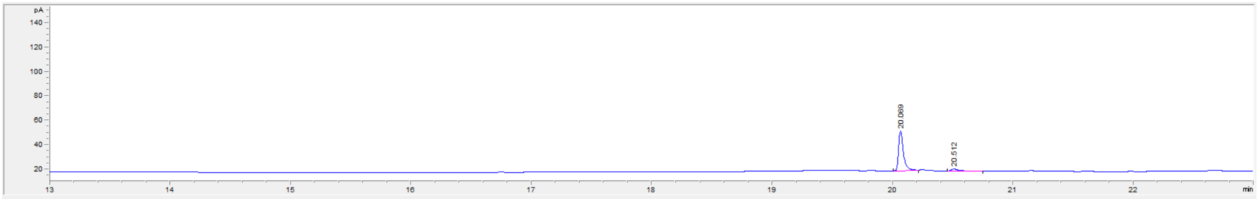

(*S*)-2-(4-methylphenyl)propanoic acid ((*S*)-**3e**) from biotransformation

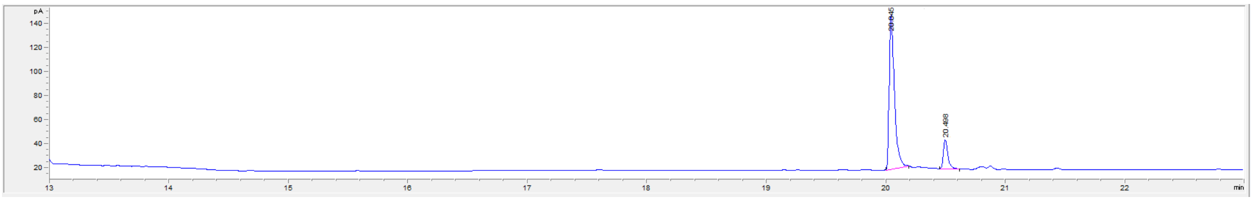

Analytical data for 2-(4-methoxyphenyl)propanal (1f)

Column: RESTEK Rt-βDEXsm (30 m x 0.32 mm x 0.25 µm)
Temperature program: 100 °C, hold 0 min; 10 °C/min to 130 °C, hold 20 min; 2 °C/min to 140 °C, hold 3 min; 5 °C/min to 150 °C, hold 0 min; 10 °C/min to 220 °C, hold 1 min.
Retention times: t_R (1) = 19.6 min, t_R (2) = 20.0 min

Analytical data for 2-(4-methoxyphenyl)propan-1-ol (2f)

Column: RESTEK Rt-βDEXsm (30 m x 0.32 mm x 0.25 µm)
Temperature program: 100 °C, hold 0 min; 10 °C/min to 130 °C, hold 20 min; 2 °C/min to 140 °C, hold 3 min; 5 °C/min to 150 °C, hold 0 min; 10 °C/min to 220 °C, hold 1 min.
Retention times: t_R (*R*) = 29.7 min, t_R (*S*) = 30.2 min

Reference *rac*-2-(4-methoxyphenyl)propan-1-ol (**2f**)

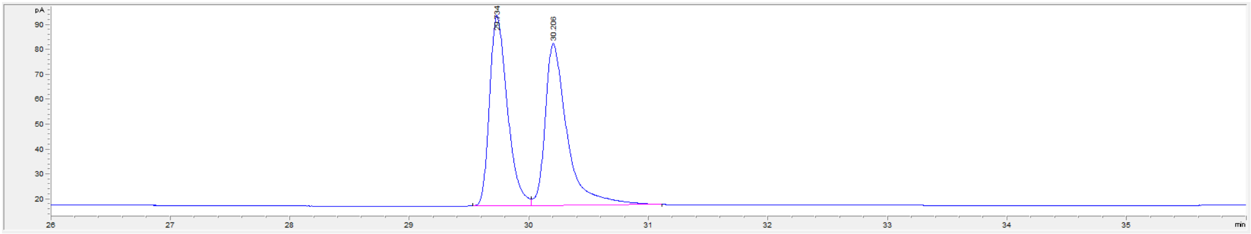

Reference (*S*)-2-(4-methoxyphenyl)propan-1-ol ((*S*)-**2f**)

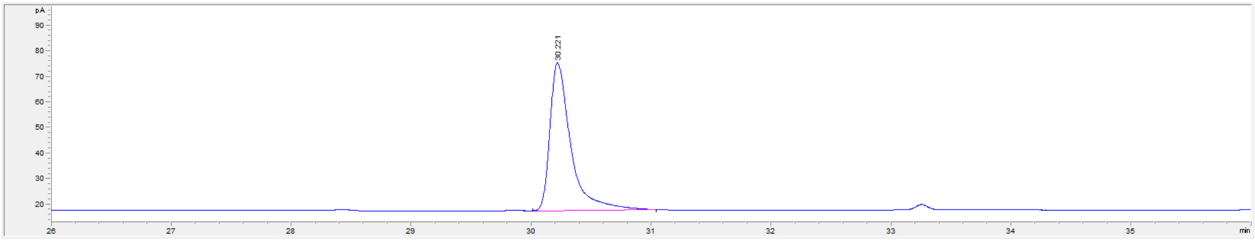

(*S*)-2-(4-methoxyphenyl)propan-1-ol ((*S*)-**2f**) from biotransformation

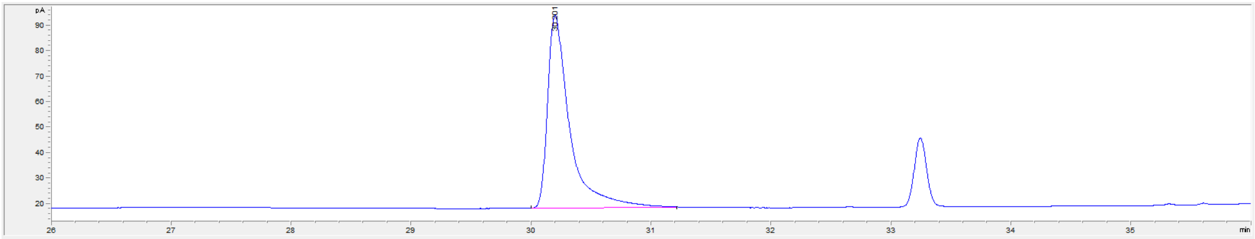

Analytical data for 2-(4-methoxyphenyl)propanoic acid (3f)

Column: Agilent CP-Chirasil-DEX CB column (25 m x 0.32 mm x 0.25 µm)
Temperature program: 100 °C, hold 1 min; 5 °C/min to 140 °C, hold 3 min; 10 °C/min to 180 °C, hold 8 min.
Retention times: t_R (*S*) = 19.3 min, t_R (*R*) = 19.9 min

Reference *rac*-2-(4-methoxyphenyl)propanoic acid (**3f**)

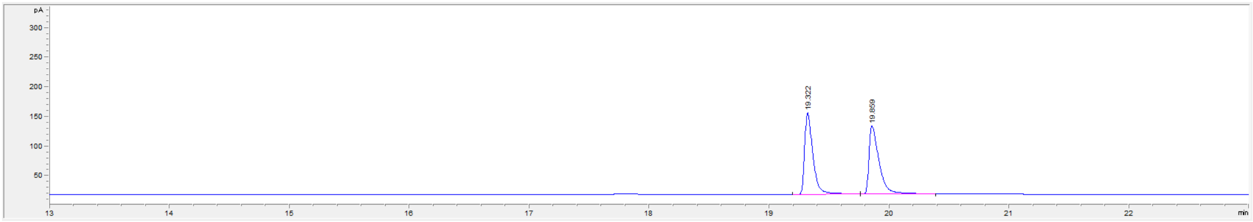

Reference (*S*)-2-(4-methoxyphenyl)propanoic acid ((*S*)-**3f**)

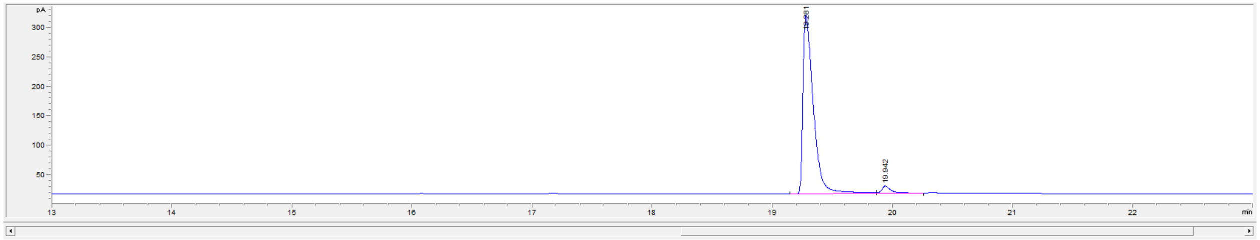

Reference (*S*)-2-(4-methoxyphenyl)propanoic acid ((*S*)-**3f**) from biotransformation

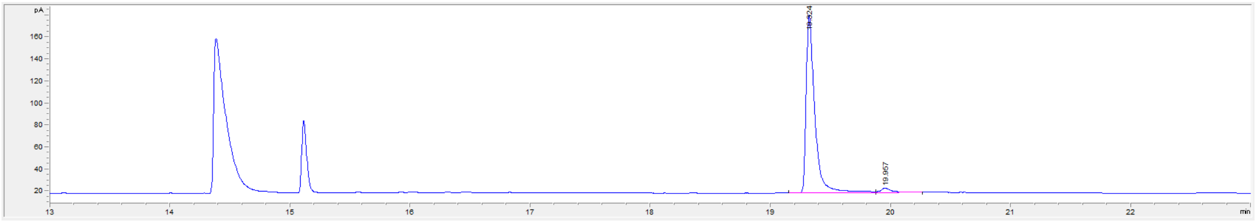

Analytical data 2-(4-isobutylphenyl)propanal (1g)

Column: RESTEK Rt-βDEXsm (30 m x 0.32 mm x 0.25 µm)
Temperature program: 100 °C, hold 0 min; 10 °C/min to 120 °C, hold 25 min; 2 °C/min to 150 °C, hold 0 min; 10 °C/min to 220 °C, hold 1 min.
Retention times: t_R (1) = 34.3 min, t_R (2) = 34.5 min

Analytical data 2-(4-isobutylphenyl)propan-1-ol (2g)

Column: RESTEK Rt-βDEXsm (30 m x 0.32 mm x 0.25 µm)
Temperature program: 100 °C, hold 0 min; 10 °C/min to 120 °C, hold 25 min; 2 °C/min to 150 °C, hold 0 min; 10 °C/min to 220 °C, hold 1 min.
Retention times: t_R (*R*) = 42.1 min, t_R (*S*) = 42.4 min

Reference *rac*-2-(4-isobutylphenyl)propan-1-ol (**2g**)

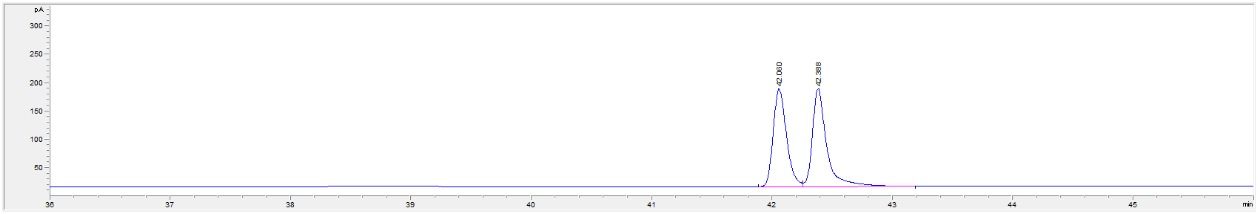

Reference (*S*)-2-(4-isobutylphenyl)propan-1-ol ((*S*)-**2g**)

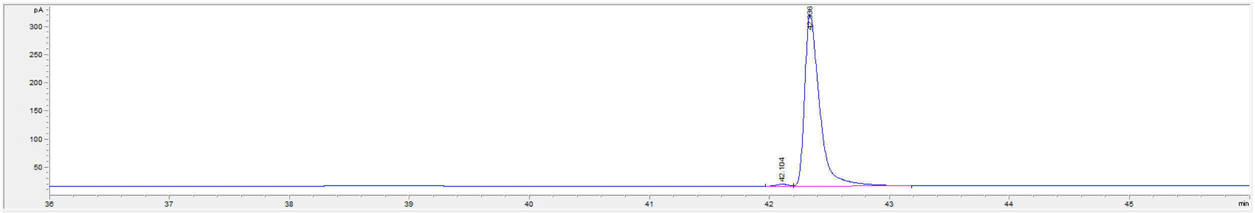

(*S*)-2-(4-isobutylphenyl)propan-1-ol ((*S*)-**2g**) from biotransformation

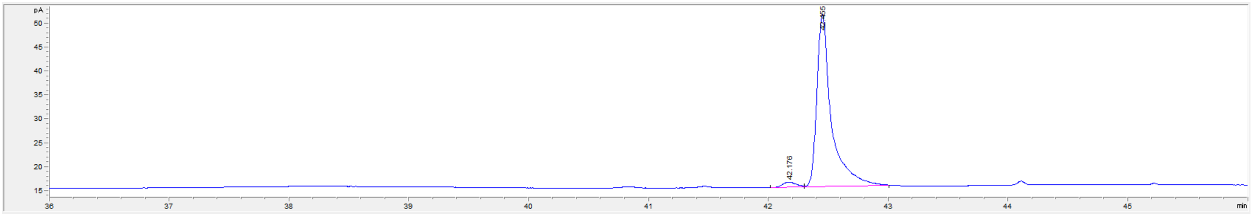

Analytical data for 2-(4-isobutylphenyl)propanoic acid (3g)

Column: Agilent CP-Chirasil-DEX CB column (25 m x 0.32 mm x 0.25 µm)
Temperature program: 100 °C, hold 0.5 min; 5 °C/min to 180 °C, hold 6 min; 10 °C/min to 200 °C, hold 1 min.
Retention times: t_R (*S*) = 19.6 min, t_R (*R*) = 20.3 min

Reference *rac*-2-(4-isobutylphenyl)propanoic acid (**3g**) purchased

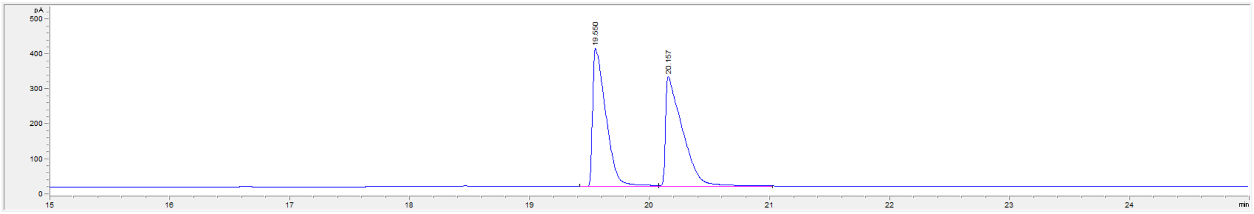

Reference (*S*)-2-(4-isobutylphenyl)propanoic acid ((*S*)-**3g**) purchased

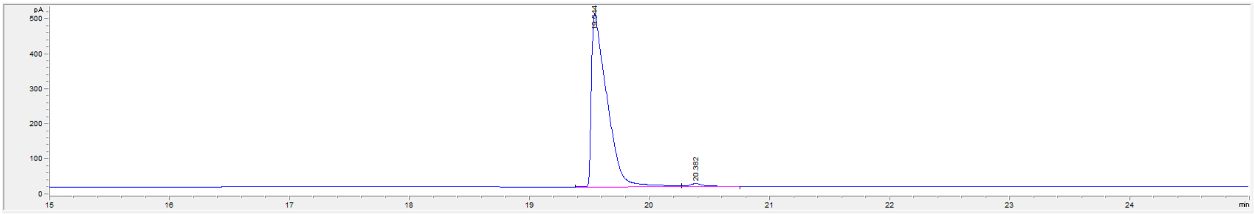

(*S*)-2-(4-isobutylphenyl)propanoic acid ((*S*)-**3g**) from biotransformation

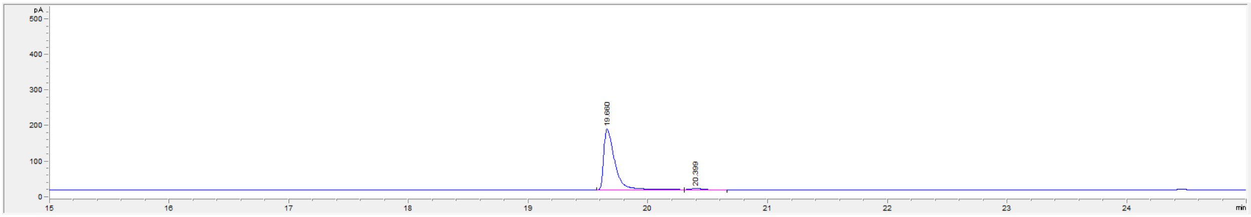

7. References

1. Clark, W.; Still, W. C.; Kahn, M.; Mitra, A., *J. Org. Chem.* **1978**, 43 (14), 2923-2925.
2. Fuchs, C. S.; Hollauf, M.; Meissner, M.; Simon, R. C.; Besset, T.; Reek, J. N. H.; Riethorst, W.; Zepeck, F.; Kroutil, W., *Adv. Synth. Catal.* **2014**, 356 (10), 2257-2265.
3. Theodorou, A.; Papadopoulos, G. N.; Kokotos, C. G., *Tetrahedron* **2013**, 69 (26), 5438-5443.
4. Robinson, M. W. C.; Pillinger, K. S.; Mabbett, I.; Timms, D. A.; Graham, A. E., *Tetrahedron* **2010**, 66 (43), 8377-8382.

5. Friest, J. A.; Maezato, Y.; Broussy, S.; Blum, P.; Berkowitz, D. B., *J. Am. Chem. Soc.* **2010**, 132 (17), 5930-5931.
6. Gansauer, A.; Klatte, M.; Brandle, G. M.; Friedrich, J., *Angew. Chem. Int. Ed.* **2012**, 51 (35), 8891-8894.
7. Strübing, D.; Krumlinde, P.; Piera, J.; Bäckvall, J.-E., *Adv. Synth. Catal.* **2007**, 349 (10), 1577-1581.
8. Serra, S., *Tetrahedron: Asymmetry* **2011**, 22 (6), 619-628.
9. Greenhalgh, M. D.; Thomas, S. P., *J. Am. Chem. Soc.* **2012**, 134 (29), 11900-11903.
10. León, T.; Correa, A.; Martin, R., *J. Am. Chem. Soc.* **2013**, 135 (4), 1221-1224.
11. Müller, T.; Johann, L.; Jannack, B.; Brückner, M.; Lanfranchi, D. A.; Bauer, H.; Sanchez, C.; Yardley, V.; Deregnaucourt, C.; Schrével, J.; Lanzer, M.; Schirmer, R. H.; Davioud-Charvet, E., *J. Am. Chem. Soc.* **2011**, 133 (30), 11557-11571.
12. Galletti, P.; Emer, E.; Gucciardo, G.; Quintavalla, A.; Pori, M.; Giacomini, D., *Org. Biomol. Chem.* **2010**, 8 (18), 4117-4123.
13. Allmendinger, S.; Kinuta, H.; Breit, B., *Adv. Synth. Catal.* **2015**, 357 (1), 41-45.
14. Guduguntla, S.; Fañanás-Mastral, M.; Feringa, B. L., *J. Org. Chem.* **2013**, 78 (17), 8274-8280.
15. Shvartsbart, A.; Smith, A. B., *J. Am. Chem. Soc.* **2015**, 137 (10), 3510-3519.
16. Jackson, J. J.; Kobayashi, H.; Steffens, S. D.; Zakarian, A., *Angew. Chem. Int. Ed.* **2015**, 54 (34), 9971-9975.
17. Jiang, Y.; Chen, C. A.; Lu, K.; Daniewska, I.; De Leon, J.; Kong, R.; Forray, C.; Li, B.; Hegde, L. G.; Wolinsky, T. D.; Craig, D. A.; Wetzel, J. M.; Andersen, K.; Marzabadi, M. R., *J. Med. Chem.* **2007**, 50 (16), 3870-3882.
18. Wu, Z.-L.; Li, Z.-Y., *Tetrahedron: Asymmetry* **2001**, 12 (23), 3305-3312.
19. Jang, H.; Jung, B.; Hoveyda, A. H., *Org. Lett.* **2014**, 16 (17), 4658-4661.

8. ^{1}H NMR spectra

2-phenylpropanol (**2a**) isolated from preparative scale reaction (10 mL)

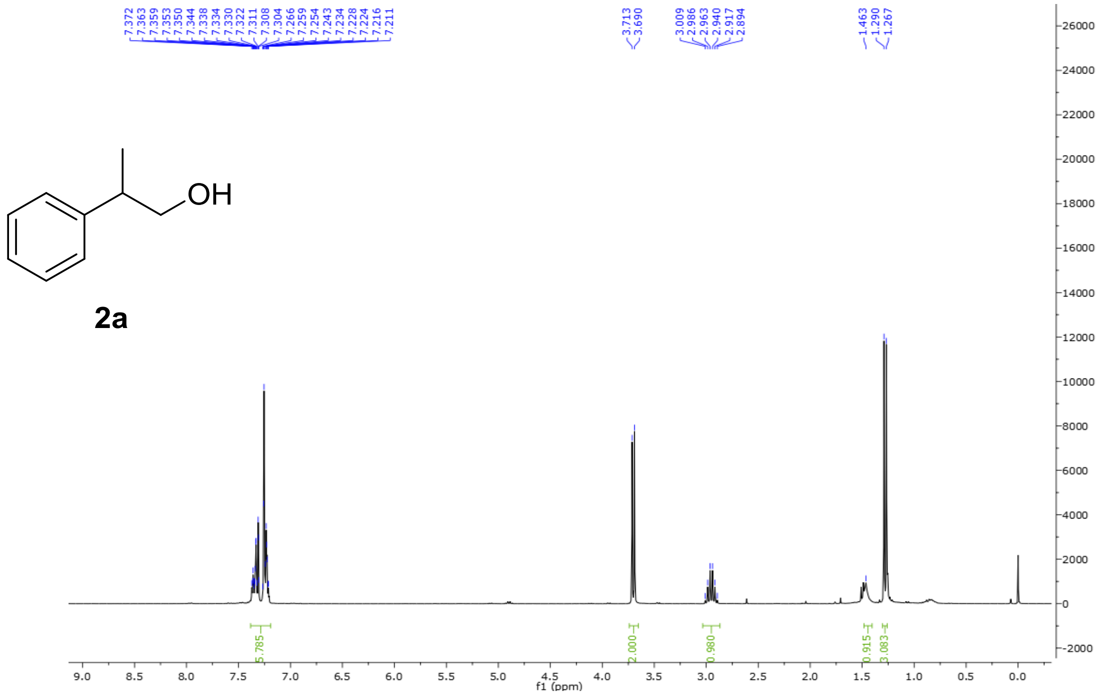

2-phenylpropanoic acid (**3a**) isolated from preparative scale reaction (10 mL)

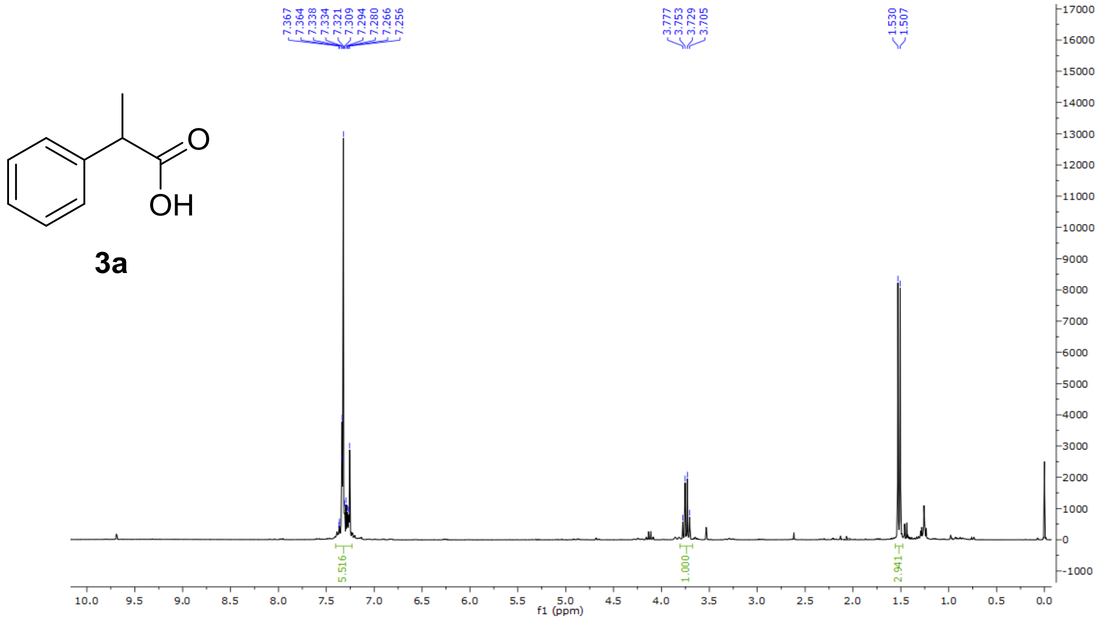

2-(4-fluorophenyl)propanal (**1b**)

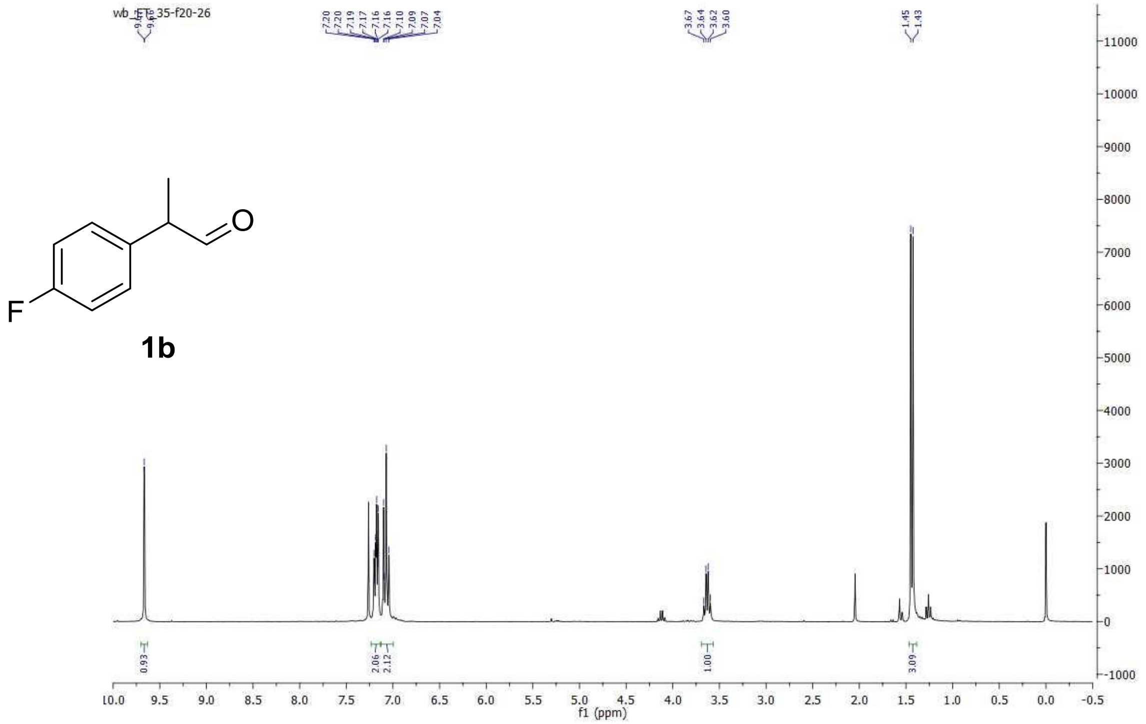

2-(4-(trifluoromethyl)phenyl)propanal (**1c**)

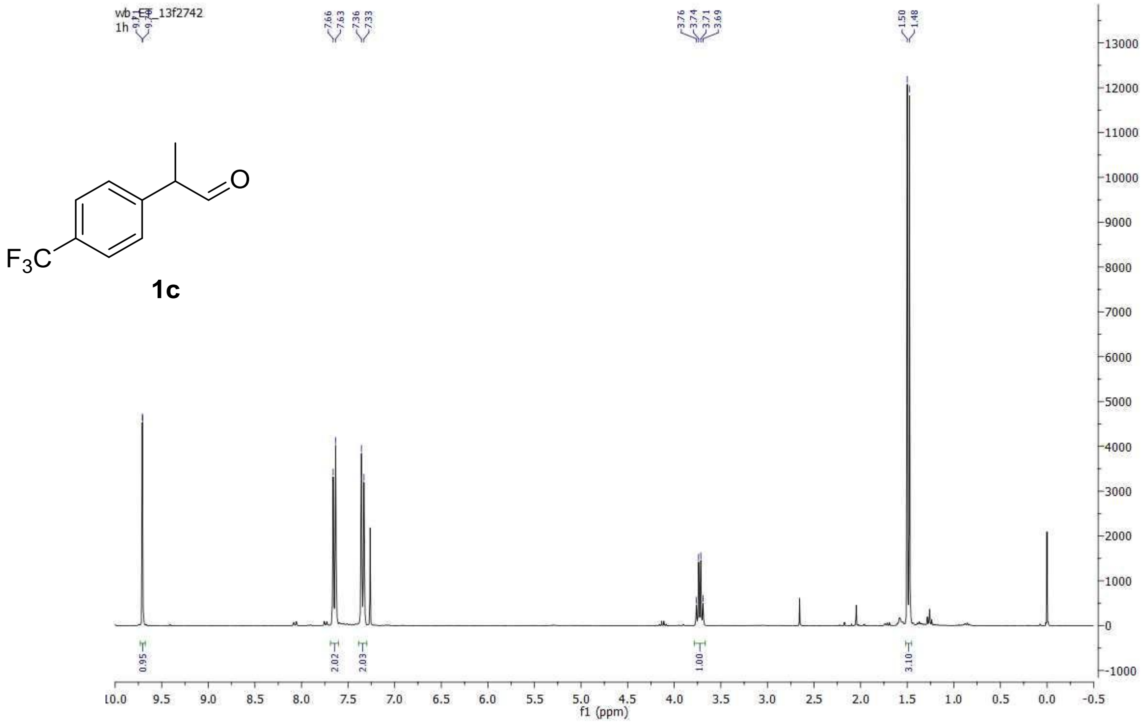

2-(4-bromophenyl)propanal (**1d**)

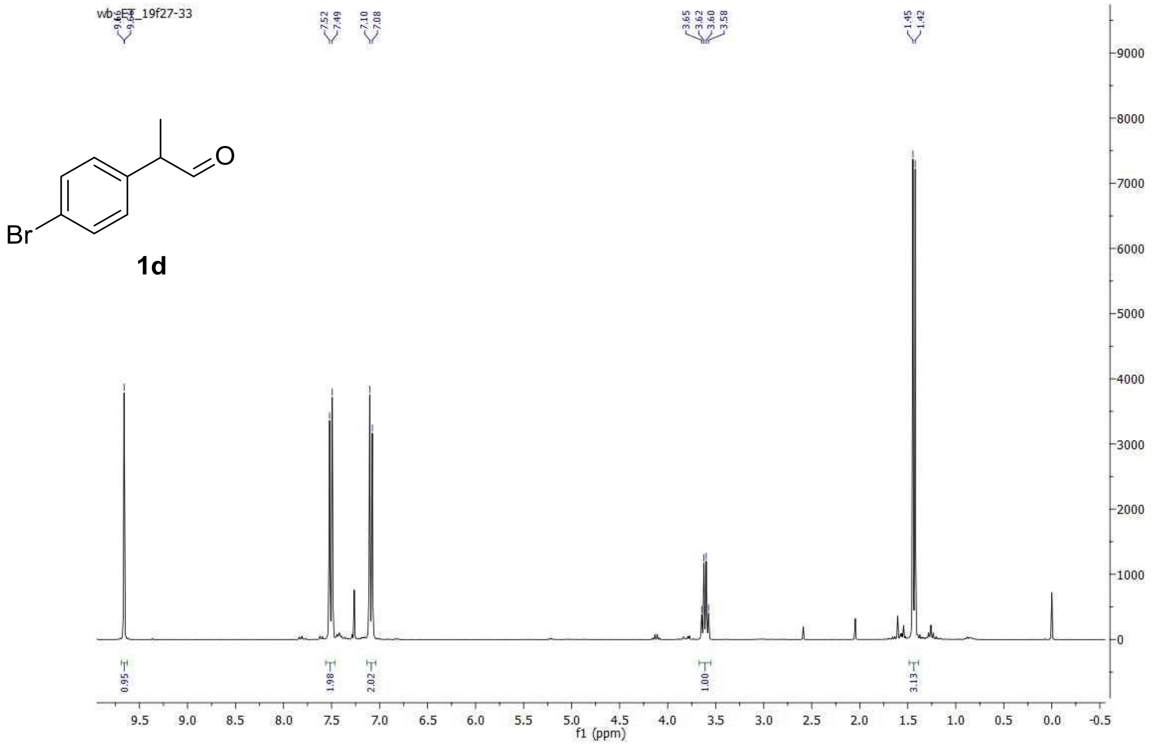

2-(4-methylphenyl)propanal (**1e**)

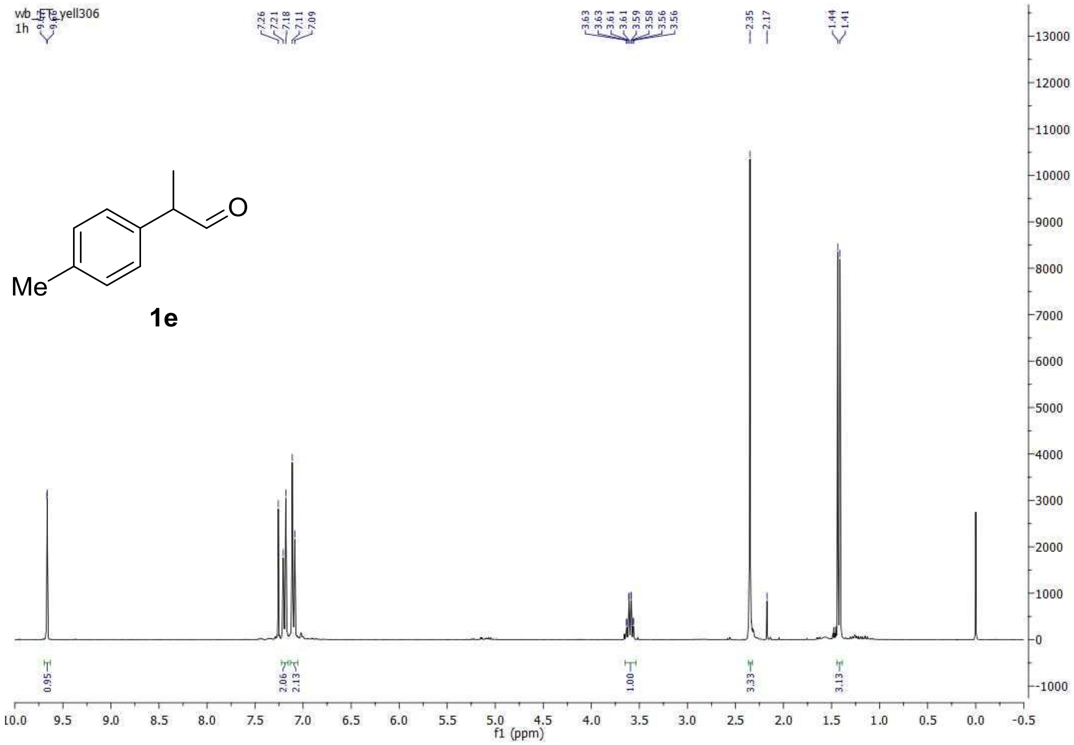

2-(4-methoxyphenyl)propanal (**1f**)

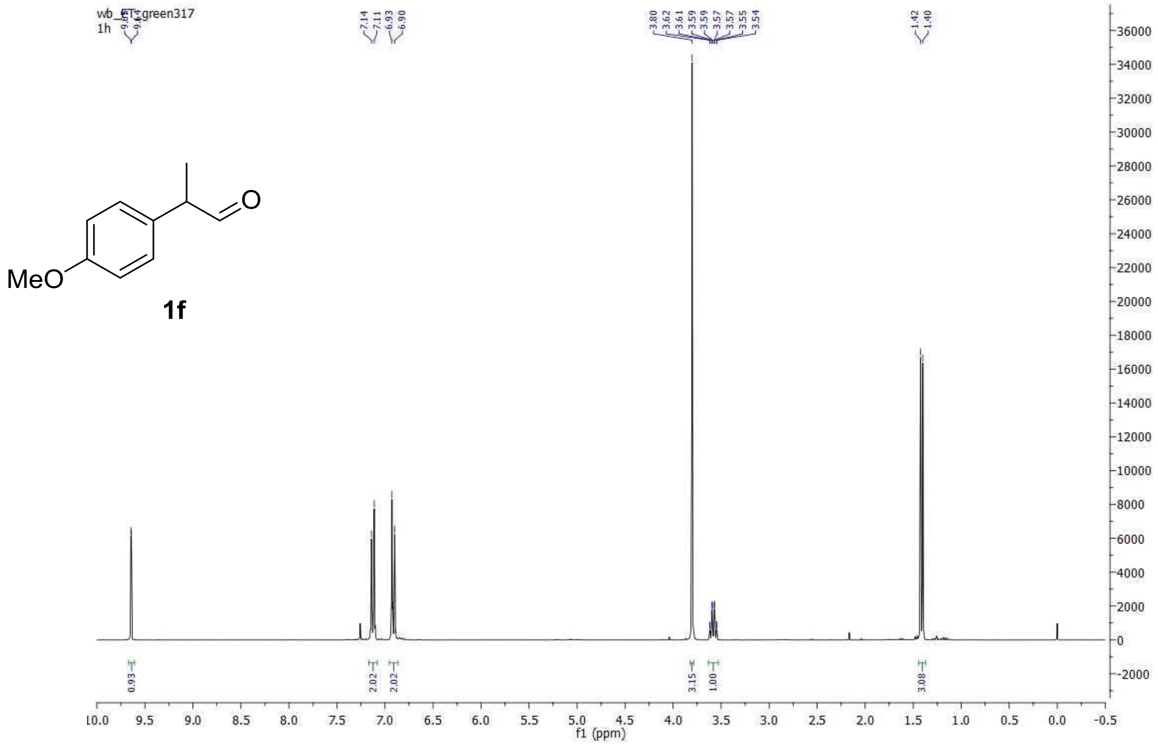

2-(4-isobutylphenyl)propanal (**1g**)

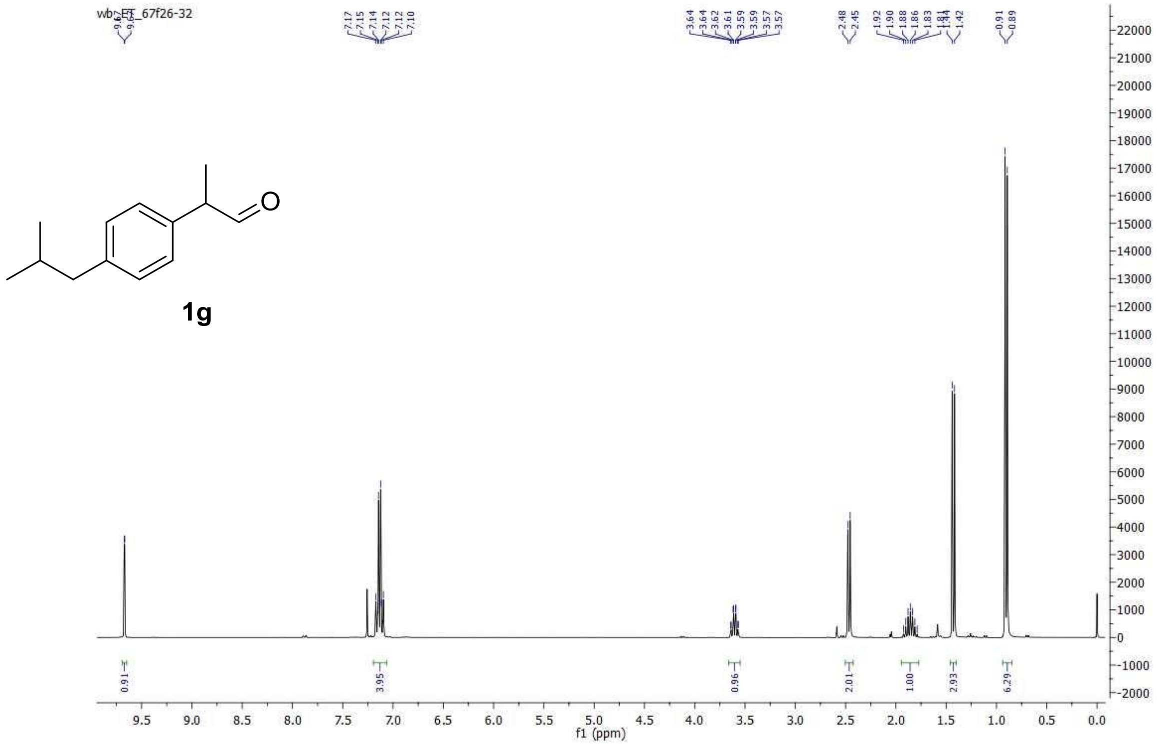

2-(4-fluorophenyl)propan-1-ol (**2b**)

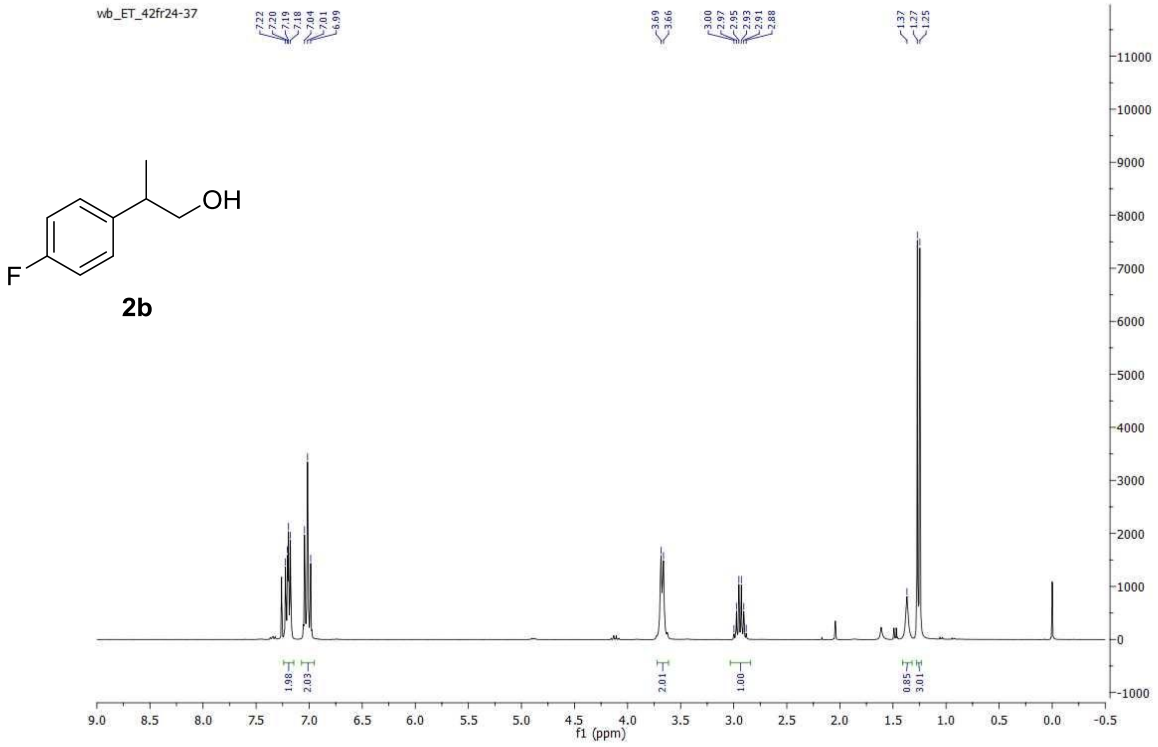

2-(4-(trifluoromethyl)phenyl)propan-1-ol (**2c**)

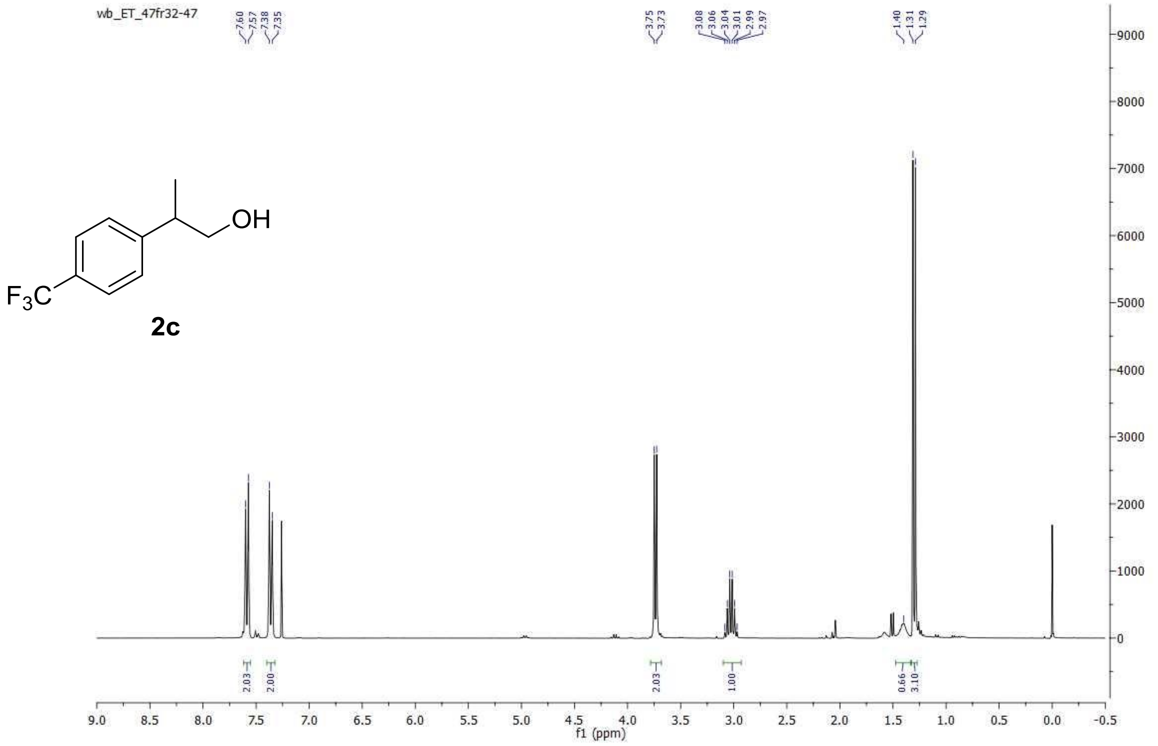

2-(4-bromophenyl)propan-1-ol (**2d**)

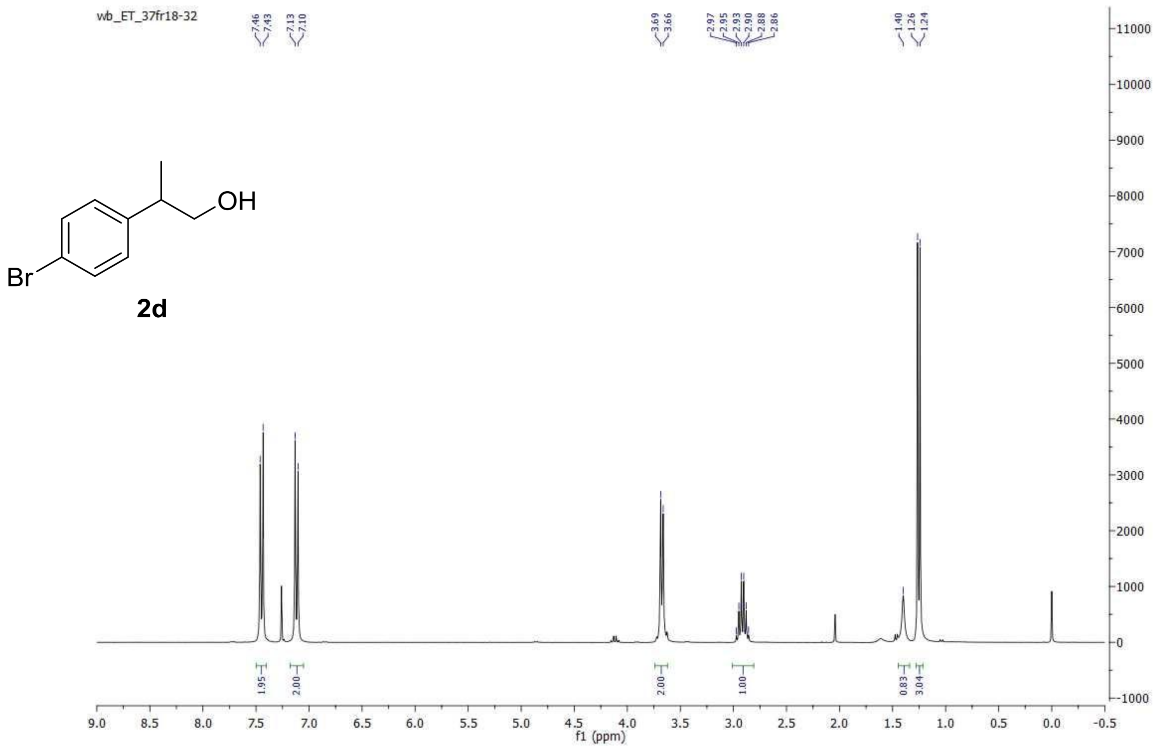

2-(4-methylphenyl)propan-1-ol (**2e**)

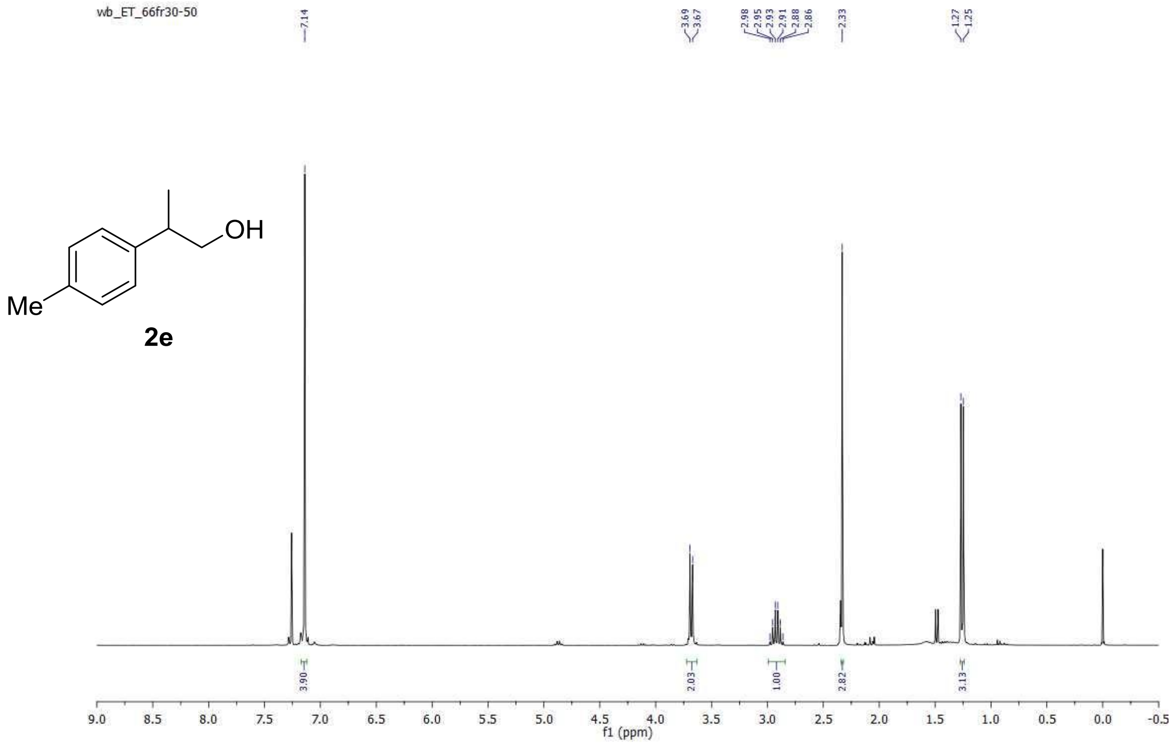

2-(4-methoxyphenyl)propan-1-ol (**2f**)

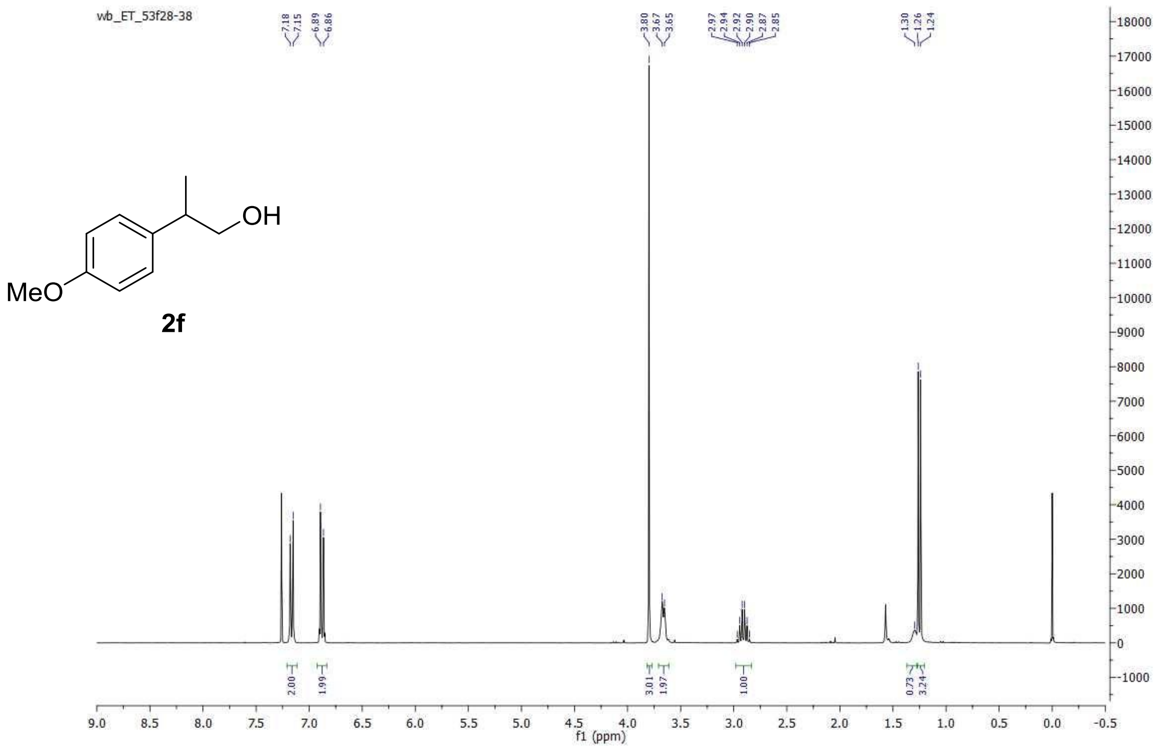

2-(4-isobutylphenyl)propan-1-ol (**2g**)

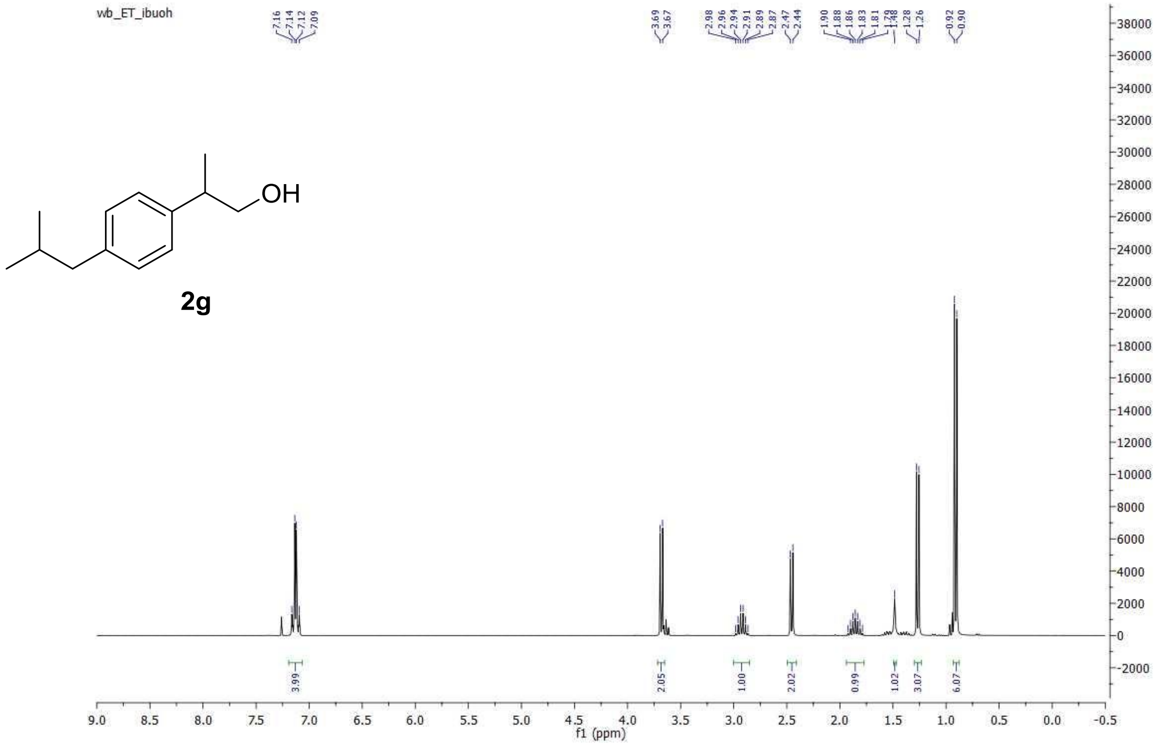

2-(4-fluorophenyl)propanoic acid (**3b**)

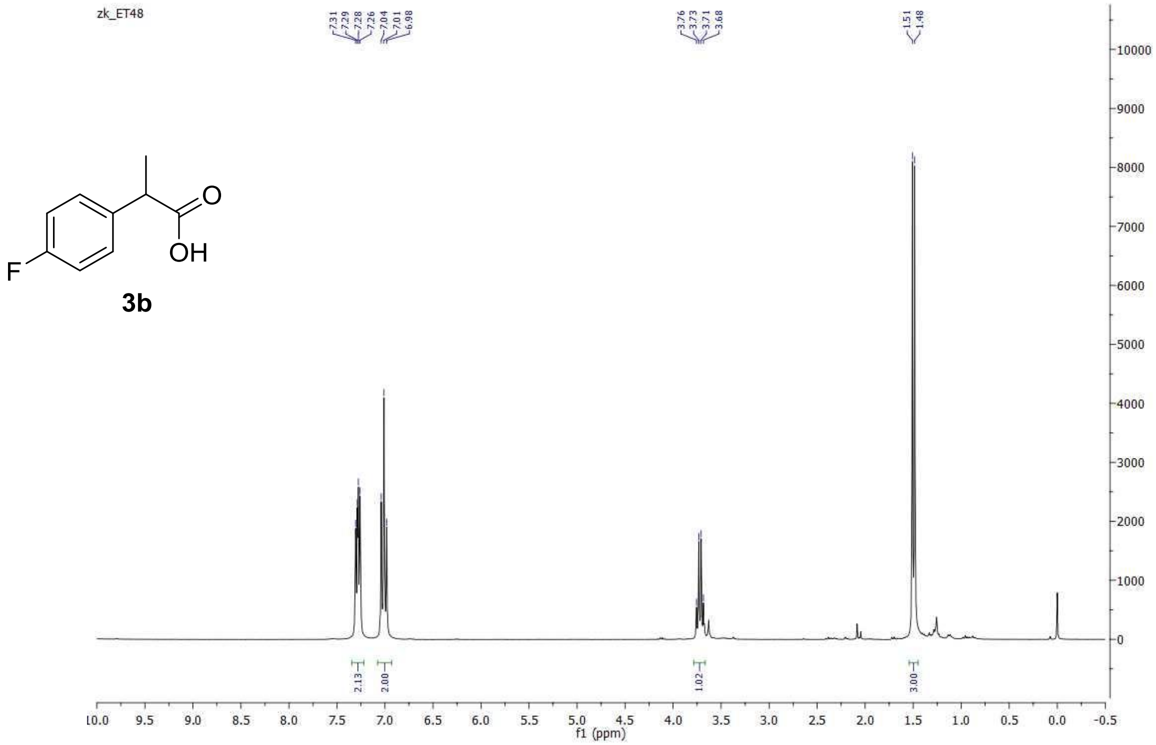

2-(4-(trifluoromethyl)phenyl)propanoic acid (**3c**)

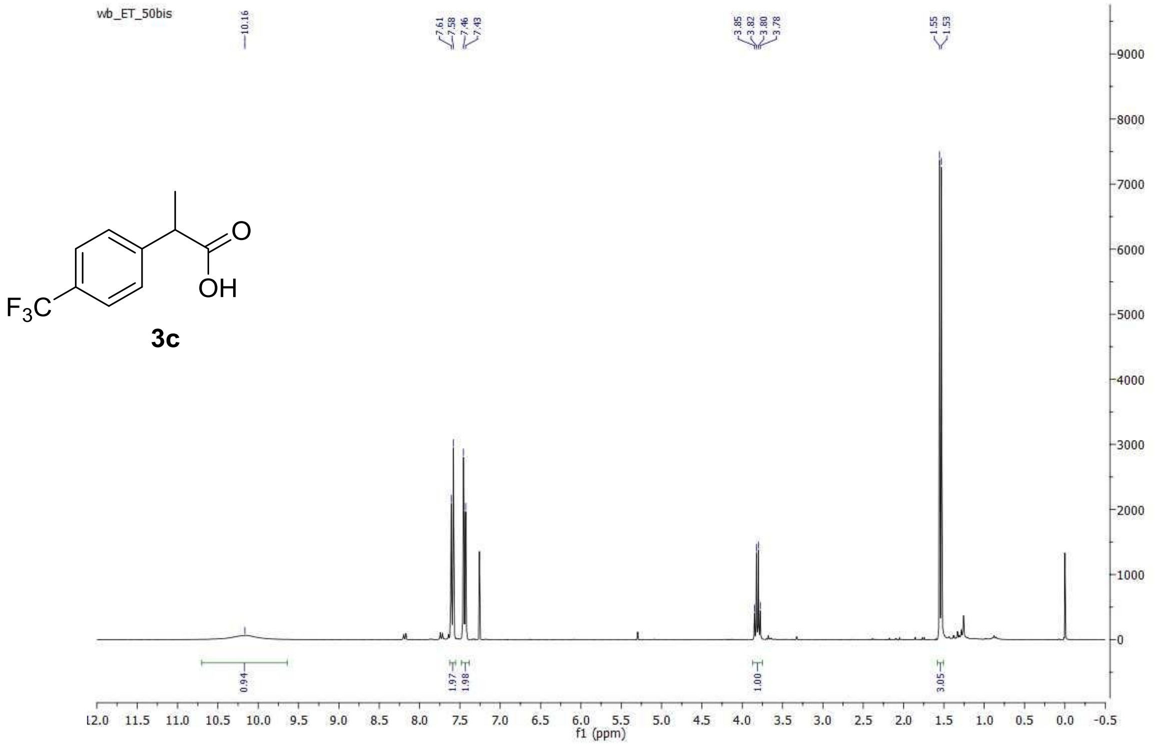

2-(4-bromophenyl)propanoic acid (**3d**)

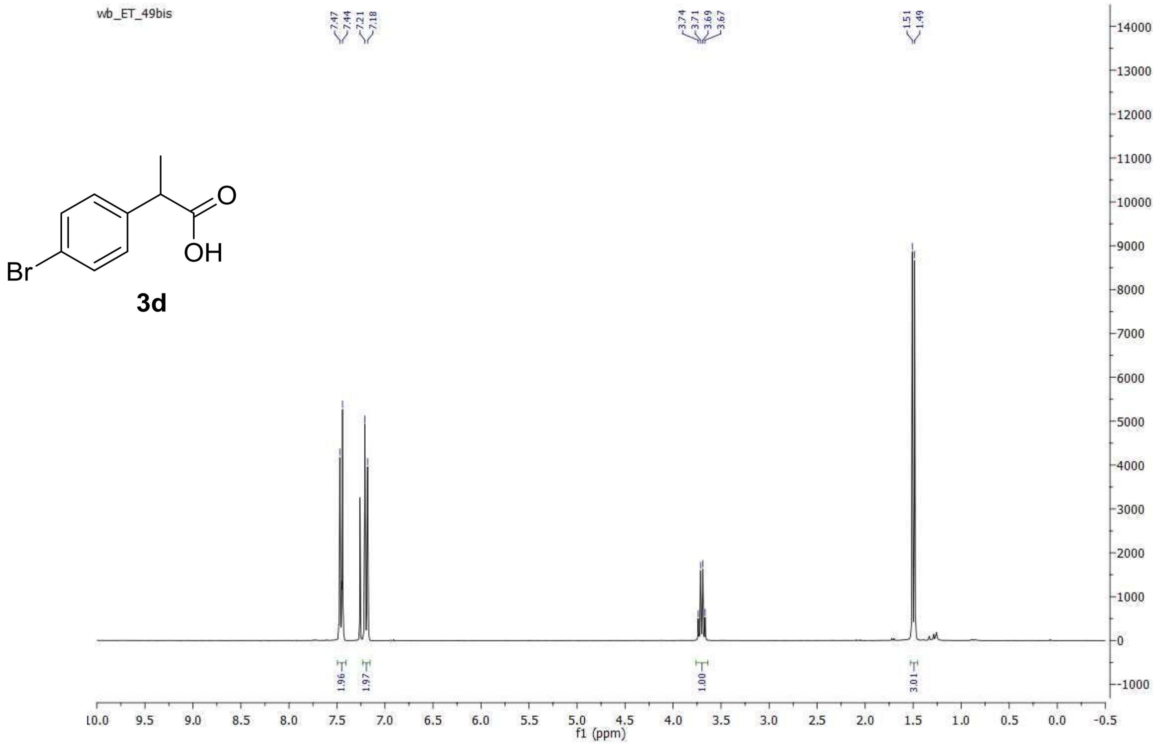

2-(4-methylphenyl)propanoic acid (**3e**)

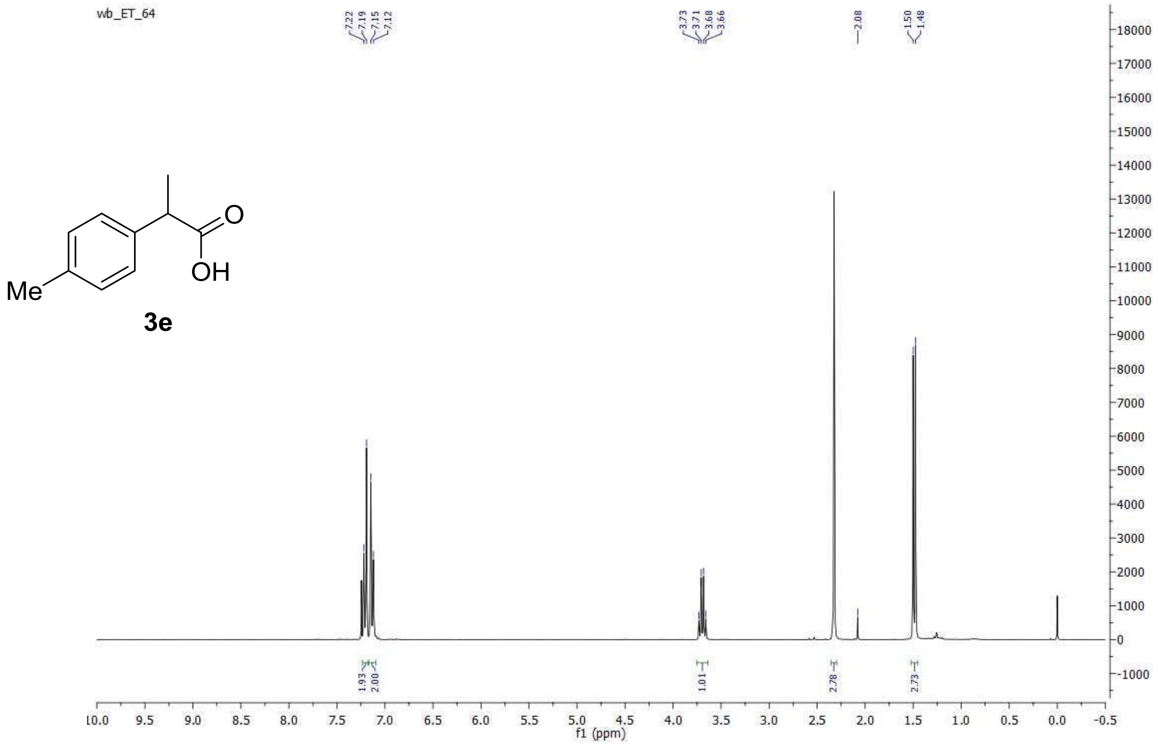

2-(4-methoxyphenyl)propanoic acid (**3f**)

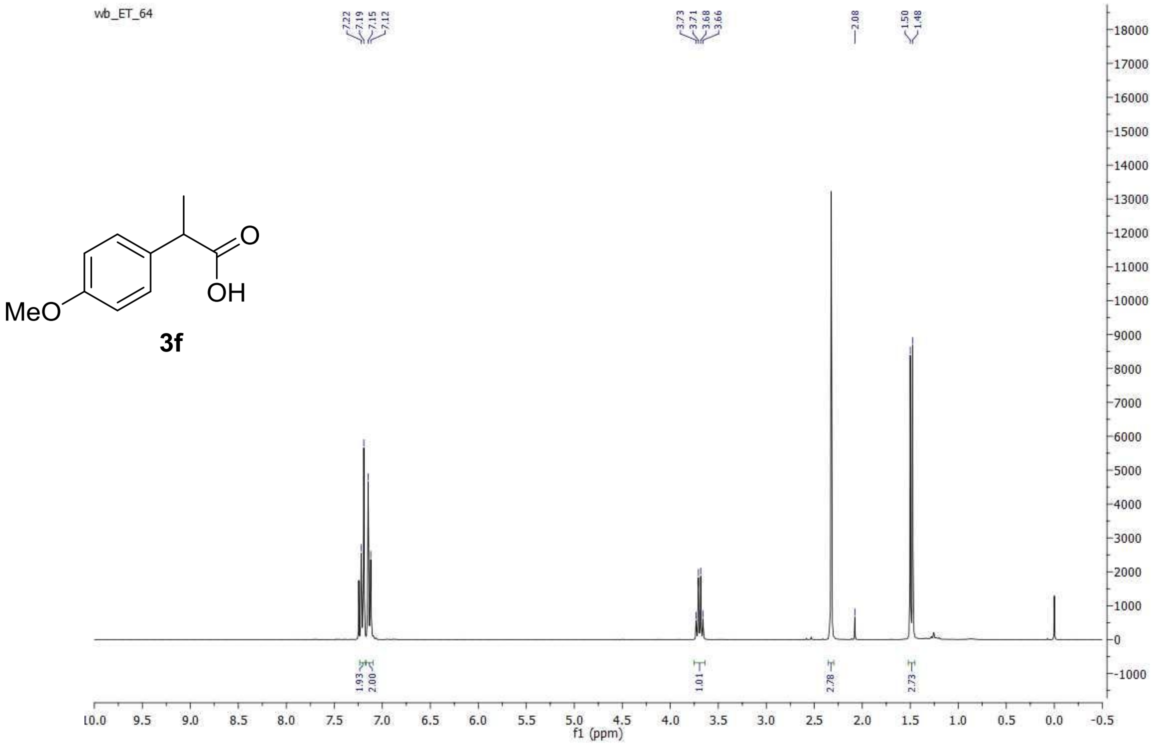

Supplement: Supplementary file 1 — Supplementary [file ADSC-360-2742-s001.pdf]
